# Selective serotonin reuptake inhibitors versus placebo in patients with major depressive disorder. A systematic review with meta-analysis and Trial Sequential Analysis

**DOI:** 10.1186/s12888-016-1173-2

**Published:** 2017-02-08

**Authors:** Janus Christian Jakobsen, Kiran Kumar Katakam, Anne Schou, Signe Gade Hellmuth, Sandra Elkjær Stallknecht, Katja Leth-Møller, Maria Iversen, Marianne Bjørnø Banke, Iggiannguaq Juhl Petersen, Sarah Louise Klingenberg, Jesper Krogh, Sebastian Elgaard Ebert, Anne Timm, Jane Lindschou, Christian Gluud

**Affiliations:** 1 The Copenhagen Trial Unit, Centre for Clinical Intervention Research, Department 7812 Rigshospitalet, Copenhagen University Hospital, Blegdamsvej 9, Rigshospitalet, DK 2100 Copenhagen, Denmark; 2 Department of Cardiology, Holbæk Hospital, Holbæk, Denmark; 3 Mental Health Centre Copenhagen, Faculty of Health Sciences, University of Copenhagen, Copenhagen, Denmark

**Keywords:** Depression, SSRI, Systematic review

## Abstract

**Background:**

The evidence on selective serotonin reuptake inhibitors (SSRIs) for major depressive disorder is unclear.

**Methods:**

Our objective was to conduct a systematic review assessing the effects of SSRIs versus placebo, ‘active’ placebo, or no intervention in adult participants with major depressive disorder. We searched for eligible randomised clinical trials in The Cochrane Library’s CENTRAL, PubMed, EMBASE, PsycLIT, PsycINFO, Science Citation Index Expanded, clinical trial registers of Europe and USA, websites of pharmaceutical companies, the U.S. Food and Drug Administration (FDA), and the European Medicines Agency until January 2016. All data were extracted by at least two independent investigators. We used Cochrane systematic review methodology, Trial Sequential Analysis, and calculation of Bayes factor. An eight-step procedure was followed to assess if thresholds for statistical and clinical significance were crossed. Primary outcomes were reduction of depressive symptoms, remission, and adverse events. Secondary outcomes were suicides, suicide attempts, suicide ideation, and quality of life.

**Results:**

A total of 131 randomised placebo-controlled trials enrolling a total of 27,422 participants were included. None of the trials used ‘active’ placebo or no intervention as control intervention. All trials had high risk of bias. SSRIs significantly reduced the Hamilton Depression Rating Scale (HDRS) at end of treatment (mean difference −1.94 HDRS points; 95% CI −2.50 to −1.37; *P* < 0.00001; 49 trials; Trial Sequential Analysis-adjusted CI −2.70 to −1.18); Bayes factor below predefined threshold (2.01*10^−23^). The effect estimate, however, was below our predefined threshold for clinical significance of 3 HDRS points. SSRIs significantly decreased the risk of no remission (RR 0.88; 95% CI 0.84 to 0.91; *P* < 0.00001; 34 trials; Trial Sequential Analysis adjusted CI 0.83 to 0.92); Bayes factor (1426.81) did not confirm the effect). SSRIs significantly increased the risks of serious adverse events (OR 1.37; 95% CI 1.08 to 1.75; *P* = 0.009; 44 trials; Trial Sequential Analysis-adjusted CI 1.03 to 1.89). This corresponds to 31/1000 SSRI participants will experience a serious adverse event compared with 22/1000 control participants. SSRIs also significantly increased the number of non-serious adverse events. There were almost no data on suicidal behaviour, quality of life, and long-term effects.

**Conclusions:**

SSRIs might have statistically significant effects on depressive symptoms, but all trials were at high risk of bias and the clinical significance seems questionable. SSRIs significantly increase the risk of both serious and non-serious adverse events. The potential small beneficial effects seem to be outweighed by harmful effects.

**Systematic review registration:**

PROSPERO CRD42013004420.

**Electronic supplementary material:**

The online version of this article (doi:10.1186/s12888-016-1173-2) contains supplementary material, which is available to authorized users.

## Background

Selective serotonin reuptake inhibitors (SSRIs) are often first-line treatment for depression and prescriptions for SSRIs are increasing [1, 2]. A number of reviews with meta-analysis have assessed the effects of SSRIs in adults with major depressive disorder [3–8], generally concluding that SSRIs have a statistically significant effect on depressive symptoms [3–8]. However, the results of the reviews have been limited by not using predefined Cochrane methodology [3–8], only including subgroups of depressed patients [9, 10], not searching all relevant databases [3–8, 10], not systematically assessing harms [3–8, 10], and not systematically assessing risks of bias [3–8, 10]. We have summarised the characteristics and the results of previous systematic reviews in Table 1. Accordingly, the evidence on the effects of SSRIs is unclear. Using, e.g., a composite outcome of all serious adverse events (according to ICH-GCP [11]) might show how SSRIs work. Furthermore, assessments of quality of life might demonstrate if SSRIs have clinically meaningful effects. It is of utmost importance to assess the clinical significance of review results if statistically significant results are shown [12, 13].

**Table 1:** Overview of previous reviews

	First author	Title	Year of publication	Design	Type of SSRI assessed	Information sources	No. of trials	No. of patients	Published protocol	Assessment of adverse events	Assessment of risk of bias
Reviews concluding that SSRIs have beneficial effect on major depressive disorder	Gibbons et al.	Benefits From Antidepressants: Synthesis of 6-Week Patient-Level Outcomes From Double-blind Placebo-Controlled Randomized Trials of Fluoxetine and Venlafaxine	2012	Patient level meta-analysis	Flouxetine	Eli Lilly and Co	16	3595	No	No	No
	Undurraga et al.	Randomized, Placebo-Controlled Trials of Antidepressants for Acute Major Depression: Thirty-Year Meta-Analytic Review	2011	Systematic review	Fluoxetine, sertraline, paroxetine, citalopram, escitalopram	Medline, CINAH Library, Cochrane Library, PsycINFO	51	5285	No	No	Only publication bias
	Wilson et al.	Antidepressants Versus Placebo for the Depressed Elderly	2001	Cochrane review	Fluoxetine	PsycLIT, MEDLINE, EMBASE, LILACS, CINAHL, SIGLE, Psyndex, National Research Register, Dissertation Abstracts International	2	365	Yes	No	Only allocation concealment
	Aroll et al.	Antidepressants versus placebo for depression in primary care (Review)	2009	Cochrane review	Sertraline, escitalopram, citalopram	CCDANCTR	4	707	Yes	Yes	Only allocation concealment (QRS)
Reviews concluding that SSRIs have no effect on mild to moderat depression but have beneficial effect on severe depression	Fournier et al.	Antidepressant Drug Effects and Depression Severity: A Patient-Level Meta-analysis	2010	Patient level meta-analysis	Paroxetine	PubMed, PsycINFO, Cochrane Library	3	240	No	No	No
	Khan et al.	Severity of Depression and Response to Antidepressants and Placebo: An Analysis of the Food and Drug Administration Database	2002	Systematic review	Fluoxetine, sertraline, paroxetine	FDA	18	Unclear	No	No	No
Reviews concluding that SSRIs have questionable effect on major depressive disorder	Turner et al.	Selective Publication of Antidepressant Trials and Its Influence on Apparent Efficacy	2008	Systematic review	Fluoxetine, sertraline, paroxetine, citalopram, escitalopram	FDA, PubMed, Cochrane Library	38	Unclear	No	No	Only publication bias
	Kirsch et al.	Initial Severity and Antidepressant Benefits: A Meta-Analysis of Data Submitted to the Food and Drug Administration	2008	Systematic review	Fluoxetine, sertraline, paroxetine, citalopram	FDA, PubMed	21	1708	No	No	Only publication bias
Reviews not showing anything about the effects of SSRIs on major depressive disorder	Moncrieff et al.	Active Placebos Versus Antidepressants for Depression (Review)	2012	Cochrane review	No SSRIs studied, only TCAs	CCDANCTR			Yes		

Our objective was to conduct a comprehensive systematic review assessing the beneficial and harmful effects of SSRIs versus placebo, ‘active’ placebo, or no intervention in adult participants with major depressive disorder using our eight-step procedure for assessing evidence in systematic reviews [13].

## Methods

Details regarding the methodology are described in our protocol, which was registered prior to the systematic literature searches [14]. The methodology was not changed after the analysis of the review results began [14].

We included all randomised clinical trials comparing the effects of SSRIs (citalopram; escitalopram; sertraline; fluoxetine; paroxetine; or fluvoxamine) versus placebo, ‘active placebo’ (any active substance employed to mimic the adverse effects of taking a SSRI) [15], or no intervention. We also planned to perform subgroup analyses comparing the effects of the different doses (see Subgroup analyses). If a trial had three arms (e.g., a three-arm trial randomising the participants to two different SSRIs and placebo) then we divided the total number of control participants with two but kept the means and SDs in each group unchanged [16, 17].

Independent investigators searched for eligible trials published before January 2016 in The Cochrane Library’s CENTRAL, PubMed, EMBASE, PsychLIT, PsycINFO, clinicaltrials.gov., and Science Citation Index Expanded [14] (see Additional file 1: Search strategies). Trials were included irrespective of language, publication status, publication year, and publication type. To identify unpublished trials, we searched clinical trial registers of Europe and USA, websites of pharmaceutical companies, websites of U.S. Food and Drug Administration (FDA) and European Medicines Agency, and we requested the U.S. Food and Drug Administration (FDA) to provide all publicly releasable information about relevant clinical trials of SSRIs that were submitted for marketing approval.

Participants had to be 18 years or older and have a primary diagnosis of major depressive disorder based on standardised criteria, such as DSM III, DSM III-R, DSM IV, DSM V, or ICD 10 [14].

We excluded trials specifically randomising depressed participants with: a specific somatic disease, schizophrenia, or depression during or after pregnancy.

### Outcomes

#### Primary outcomes

- Depressive symptoms measured on the 17-item or 21-item Hamilton Depression Rating Scale (HDRS) [18], the Montgomery-Asberg Depression Rating Scale (MADRS) [19], or the Beck’s Depression Inventory (BDI) [20].
- Remission (Hamilton <8 points; BDI <10 points; MADRS <10 points).
- Adverse events during the intervention period which were classified as serious and non-serious adverse events [11]. Serious adverse events were defined as medical events that were life threatening, resulted in death, disability, or significant loss of function, or caused hospital admission or prolonged hospitalisation [11]. The remaining events were classified as non-serious adverse events [11].

#### Secondary outcomes

- Suicides, suicide attempts, and suicide ideation during the intervention period.
- Quality of life (scale used by the trialists).

The time point of primary interest was end of treatment (defined by trialist) [14]. We also planned to report results assessed at maximum follow-up [14].

#### Handling of missing data

If the participant was not included in the analysis of ‘no remission’, we assumed that they had ‘no remission’ [17]. If, e.g. 23/50 participants had ‘no remission’ but a total of 53 were randomised then we assumed that 26 had ‘no remission’. For all remaining outcomes we used observed data when these were reported [17].

#### Subgroup analyses

We planned the following subgroup analyses: participants with HDRS baseline ≤23 compared to >23 points; different SSRIs; trials using a placebo washout period before randomisation compared to trials using no washout period; participants with alcohol or drug dependence compared to no dependence; elderly participants compared to younger participants; duration of treatment below 8 weeks compared to equal to or above 8 weeks; and SSRI below or equal to median dose compared to above median dose.

We planned to assess if SSRIs differ according to psychiatric comorbidities [17]. Four trials included depressed participants with comorbid anxiety but none of these trials reported HDRS so this could not be performed. No other comorbidities (including borderline personality disorder, chronic depression, and treatment resistant depression) were identified in the included trials. We planned to assess if the effects of SSRIs differed: (1) when the SSRI was delivered as add-on therapy to another antidepressant drug; (2) per use of different forms of control interventions (‘active placebo’, traditional placebo, and no intervention); and (3) if electroconvulsive therapy was used as co-intervention. However, these analyses were not possible.

We used test for subgroup differences to assess if the effects of SSRIs seemed to differ between the different types of participants if either a trial specifically randomised a certain type of participants, or a trial reported results separately for each specific type of participants.

#### Selection of trials and data extraction

Review authors (KKK, AS, SGH, SES, KLM, MI, MBB, IJP, JK, SLK, AT, SE, JCJ) worked in pairs and independently selected relevant trials and extracted data. A standardised data extraction sheet was used (see Data extraction form). If a trial was identified by only one, it was discussed whether the trial should be included. In case of discrepancy, a third review author (JCJ) was consulted. We contacted review authors if relevant information was missing.

#### Assessment of the statistical and clinical significance

Our methodology was based on The Cochrane Handbook and GRADE [14, 16, 21]. We assessed statistical and clinical significance according to our eight-step procedure [13]:

1. We obtained 95% confidence intervals (95% CI) and *P*-values from all planned random-effects [22] and fixed-effect meta-analyses [16] and reported the most conservative result as the main result [13]. Review Manager version 5.3 was used for all meta-analyses [23].
2. Sensitivity analyses and subgroup analyses were conducted to explore the reasons for substantial statistical heterogeneity [13, 16]. Statistical heterogeneity was assessed by visual inspection of forest plots and by the heterogeneity (I^2^ or D^2^) [13, 16, 24, 25].
3. We defined three primary outcomes in our protocol [14]. Our threshold for significance was therefore adjusted according to problems with multiplicity [13] by dividing 0.05 with the value halfway between 1 (no adjustment) and 3 (Bonferroni adjustment) [13, 17] resulting in 0.05/2 = 0.025.
4. Cumulative meta-analyses are at risk of producing random errors due to sparse data and multiple testing of accumulating data [25, 26]. Therefore, Trial Sequential Analysis version 0.9.5.5 beta was applied to control this risk (http://www.ctu.dk/tsa/) [27]. The required information size (that is the number of participants needed in a meta-analysis to detect or reject a certain intervention effect) was calculated [25, 28]. The required information size is based upon the event proportion in the control group; the assumption of a plausible relative risk (RR) reduction; and the assumed heterogeneity or diversity of the meta-analysis [25, 29]. Trial Sequential Analysis enables testing to be conducted each time a new trial is included in the meta-analysis. Based on the required information size, trial sequential monitoring boundaries are constructed. This enables one to determine the statistical inference concerning cumulative meta-analysis that has not yet reached the required information size [25]. Firm evidence may be established if one of the trial sequential monitoring boundaries (for benefit (upper red dotted line), for harm (lower red dotted line), or for futility (vertical red dotted line)) is crossed by the cumulative Z-score before reaching the required information size, in which case further trials may turn out to be superfluous [28]. Trial Sequential Analysis- adjusted confidence intervals are also presented [28]. For dichotomous outcomes, we estimated the diversity-adjusted required information size based on the proportion of patients with an outcome in the control group, a relative risk reduction of 30%, an alpha of 2.5%, a beta of 20%, and diversity in the trials [13, 17]. For continuous outcomes, we estimated the required information size based on a HDRS mean difference of three points, the standard deviation observed in the control group, an alpha of 2.5%, a beta of 20%, and diversity in the trials [13, 17]. All outcomes were assessed with Trial Sequential Analysis [13, 24, 25].
5. We calculated Bayes factors for all primary outcomes. A low *P* value indicates that an observed result is unlikely given that there is no difference in effect between the compared intervention groups (i.e., the null hypothesis is true) [12, 13]. Even very low *P* values may, therefore, be misleading because the probability that the actual measured difference in effect of the compared interventions resulted from an a priori anticipated ‘true’ difference needs to be considered [13]. For this purpose, it is helpful to calculate Bayes factor for the primary outcomes. It will show the ratios between the *P*-value probabilities of the meta-analysis result divided by the probability of the meta-analysis result given that an anticipated intervention effect is the true effect [12, 13]. In other words, the lower the Bayes factor gets the more confident one should be that an actual intervention effect (the anticipated intervention effect) has produced the meta-analysis results and that a given difference between the compared groups is not caused by random error [12, 13]. Calculation of Bayes factor is not part of standard Cochrane methodology.
6. We assessed the potential impact of bias on the review results [16]. To assess the potential impact of missing data (incomplete outcome data bias) we assessed a ‘best-worst’ case scenario assuming that all participants lost to follow-up in the SSRI group had a beneficial outcome (the group mean plus 1 standard deviation (SD) or plus 2 SDs); and all those with missing outcomes in the placebo group have had a harmful outcome (the group mean minus 1 SD or minus 2 SDs) [13, 17]. We also performed the reverse ’worst-best-case’ scenario analysis [13, 17].
7. We assessed the risk of publication bias by visual inspection of funnel plots and by tests for funnel plot asymmetry [13, 16, 30].
8. We assessed clinical significance of our results. As previously suggested [4, 8, 31], we chose a drug-placebo difference of 3 points on the 17-item HDRS or an effect size of 0.50 SMD as the threshold for clinical significance (see Discussion) [14].

## Results

**Fig. 1:**
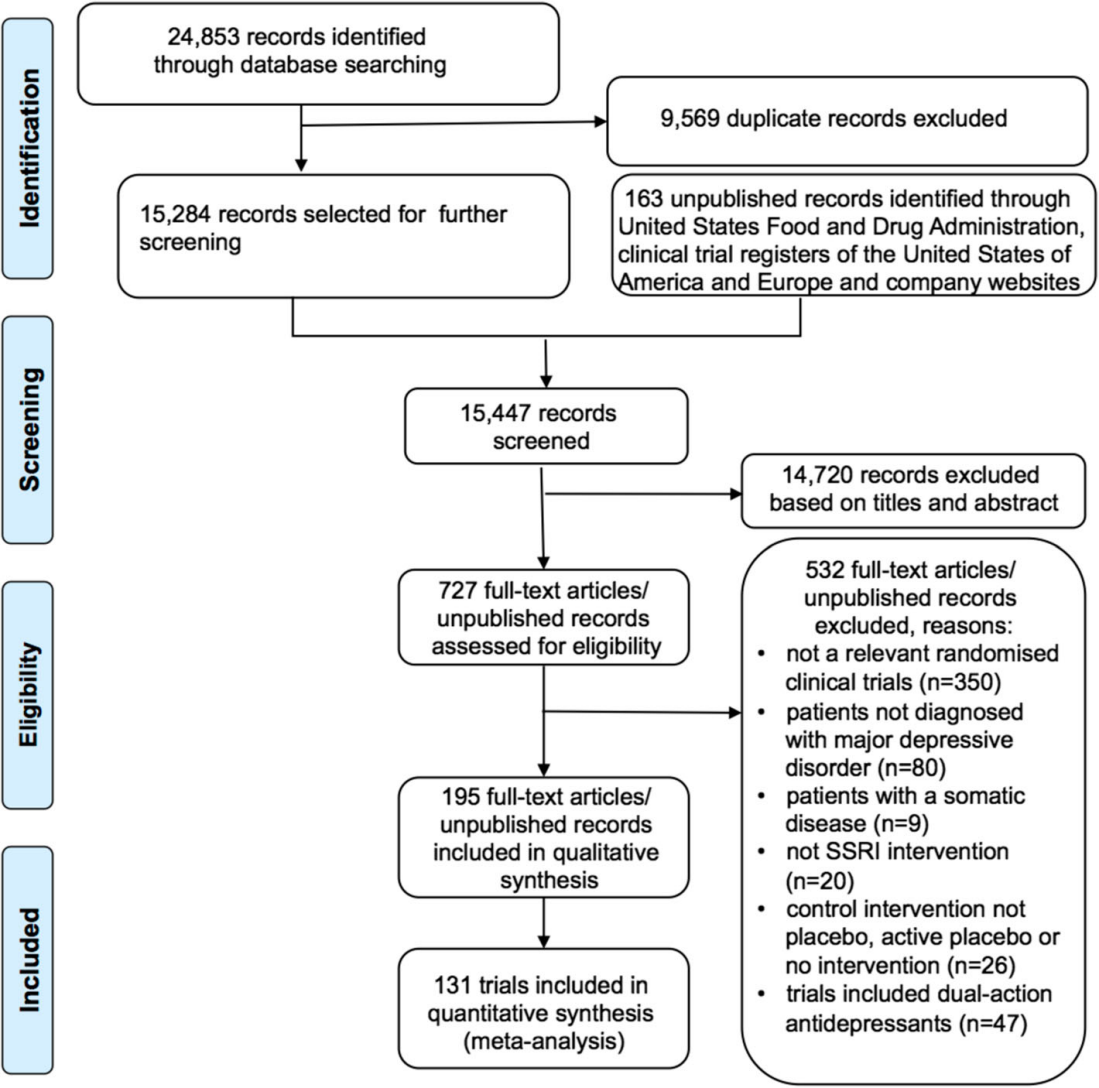
PRISMA flowchart

We have summarised the selection of trials in Fig. 1 and excluded trials in Additional file 2: List of excluded trials.

Using our strict inclusion and exclusion criteria, a total of 195 publications/unpublished trials were identified and included. Due to multiple publications of single trials and lack of useful data, only 131 trials randomised clinical trials [32–164] enrolling a total of 27,422 participants were included in our analyses. 116 were published trials [32–118, 120–131, 133–136, 141, 144, 146–148, 152, 153, 156–164] and 15 were unpublished trials [102, 119, 132, 137–140, 142, 143, 145, 149–151, 155, 165]. Of the 15 unpublished trials, eight were identified via company websites, two via clinicaltrials.gov, and four via FDA (see Additional file 3: Trial Characteristics).

We did not identify any trials using ‘active placebo’ or ‘no intervention’ as control interventions. Most trials used broad inclusion criteria and randomised adult depressed women and men below 65 years and used SSRI for 6 to 12 weeks. Twelve of the included trials specifically randomised elderly (most often participants >65 years) depressed participants [45, 48, 54, 63, 82, 110, 111, 113, 122, 144, 162, 166], five trials randomised depressed alcohol dependant participants [47, 61, 116, 120, 156], and three trials randomised depressed opioid and cocaine dependant participants [44, 115, 164]. Types of the randomised participants and other trial characteristics are summarised in Additional file 3: Trial characteristics.

### Primary outcomes

#### Hamilton depression rating scale (HDRS)

**Fig. 2:**
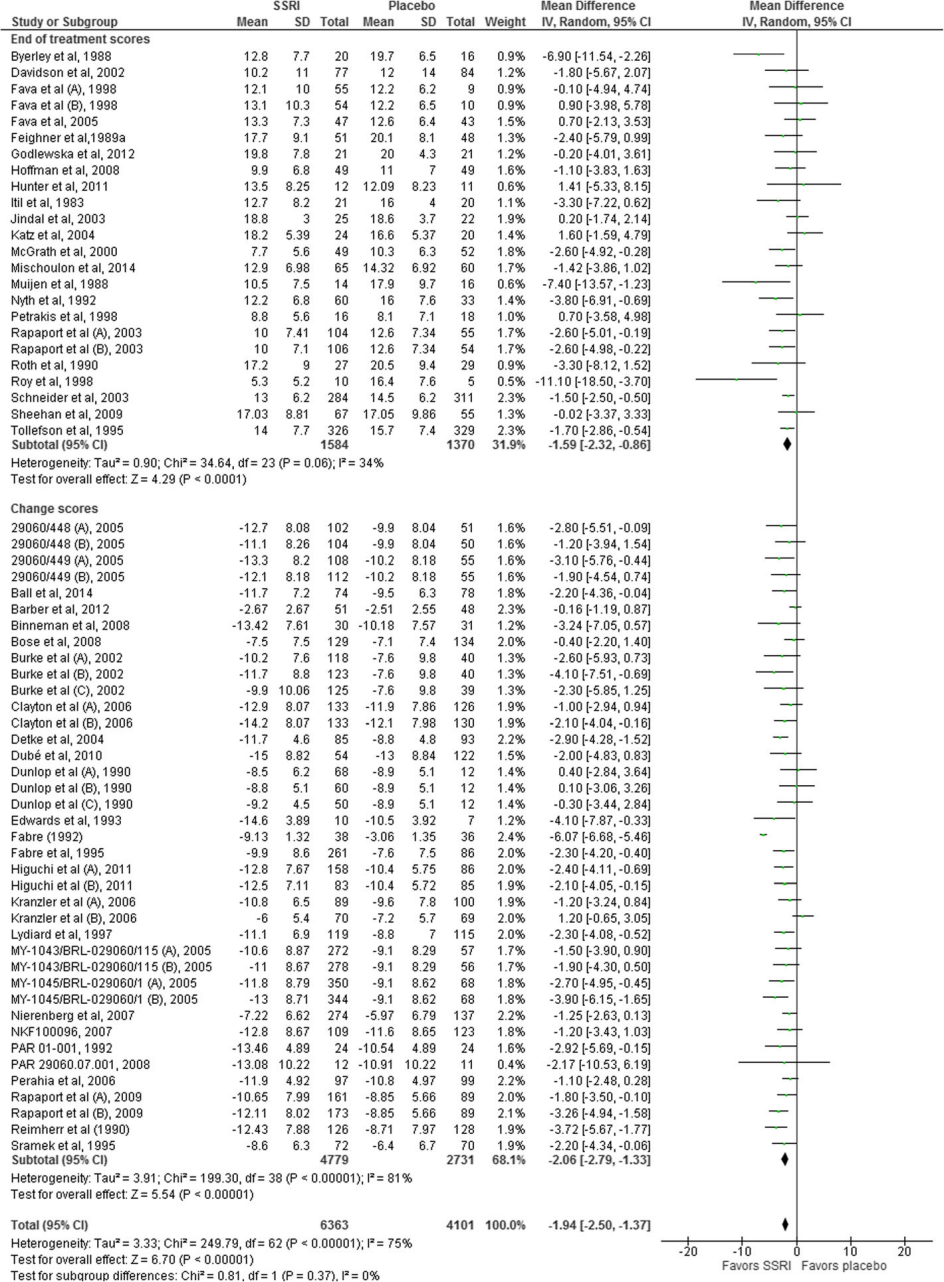
Random-effects meta-analysis of the results on the Hamilton depression rating scale

Twenty-two trials reported mean HDRS scores and standard deviation (SD) [32–50, 103, 144, 162] and 27 trials reported mean HDRS change scores and SD at end of treatment [51, 52, 54–66, 121, 123, 137, 138, 141, 142, 145, 146, 167, 168]. Random-effects meta-analysis of these 49 trials showed that SSRIs versus placebo significantly reduced the HDRS score (mean difference −1.94 points; 95% CI −2.50 to −1.37; *P* < 0.00001) (Fig. 2). Twenty-four trials reported only mean HDRS scores or presented a graph showing the mean HDRS scores, but did not report the SD at end of treatment [67–88, 166]. We planned to impute missing SDs based on observed standard deviations from trials with similar characteristics [17]. Trial characteristics, sample sizes, and statistical weight of the included trials were similar across trials and we therefore chose to impute the missing SDs with a value of 8 points (the mean of the observed standard deviations rounded up to the nearest integer). Nineteen trials reported only mean HDRS change scores or presented a graph showing the mean change HDRS scores, but did not report the SD [89–102, 108, 143, 157, 158, 169]. We imputed the missing SDs with a value of 7 points (the mean of the observed standard deviations rounded up to the nearest integer) [17]. Random-effects meta-analysis of the results of all 92 trials showed that SSRIs versus placebo significantly reduced the HDRS score (mean difference −2.25 points; 95% CI −2.69 to −1.83; *P* < 0.00001).

#### Heterogeneity

The primary meta-analysis showed statistically significant heterogeneity (*I* ^2^ = 75%; *P* < 0.00001) (Fig. 2). Visual inspection of the forest plot indicated that one trial seemed to have a more extreme effect (larger intervention effect estimate and smaller confidence interval) [58]. For exploratory purposes, we tried to exclude this trial from the analysis and this reduced the I^2^ to 29%, but removing this trial did not substantially alter the meta-analysis result (mean difference after removing the trial from the analysis −1.77 HDRS points; 95% CI −2.12 to −1.42).

#### Subgroup analysis per risk of bias

**Fig. 3:**
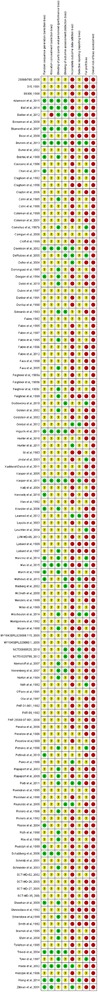
Risk of bias in the included randomized clinical trials

All the included trials had high risk of bias. Hence, it was not possible to perform a subgroup analysis of trials at low risk of bias (Fig. 3) [13, 17]. However, four trials [33, 60, 103, 121] were classified as *potentially lower* risk of bias (based on bias risk assessment of ‘generating allocation sequence’, ‘allocation concealment’, ‘blinding of participants and treatment providers’, and ‘blinding of outcome assessment’) [13, 17]. Random-effects meta-analysis of these four trials showed that SSRIs versus placebo reduced the HDRS score (mean difference −2.07 points; 95% CI −3.06 to −1.08). Test for subgroup differences between trials with *lower* risk of bias (*n* = 4) compared to trials with high risk of bias (*n* = 45) was not significant (*P* = 0.82).

#### Incomplete outcome data

Meta-analysis of the best-worst case scenario analyses adding 1 SD (mean difference −3.38 HDRS points in favour of SSRI; 95% CI −4.10 to −2.66) and adding 2 SDs (mean difference −4.50 HDRS points in favour of SSRI; 95% CI −5.37 to −3.63) for missing values showed large significant intervention effect estimates [13, 17]. Meta-analysis of the worst-best case scenario analyses showed a significant intervention effect estimate when adding 1 SD for missing values (mean difference −0.77 points; 95% CI −1.45 to −0.09) and no significant intervention effects when adding 2 SDs for missing values (mean difference 0.46 points; 95% CI −0.38 to 1.30).

#### Other subgroup analyses

Meta-analysis of the results of the 26 trials with a mean baseline HDRS score >23 points showed a mean difference of −2.69 HDRS points; 95% CI −3.59 to −1.78; *P* < 0.00001 [32, 35–38, 41, 43, 45–47, 49, 52, 57–59, 63–65, 121, 137, 142, 145, 167, 170, 171]. Meta-analysis of the results of the 20 trials with a mean baseline HDRS score ≤23 points showed a mean difference of -1.29 HDRS points; 95% CI −1.76 to −0.82; *P* < 0.00001 [33, 34, 39, 40, 44, 48, 50, 51, 54, 55, 60–63, 103, 141, 144, 146, 162, 168]. Test for subgroup difference was significant (*I* ^2^ = 86.2%; *P* = 0.007). We performed post-hoc meta-regression (STATA 14) with baseline HDRS as a covariate in the meta-analysis. This analysis showed that the effects of SSRIs seem to increase with larger baseline HDRS scores (coefficient −0.33 points; 95% CI −0.44 to −0.22; *P* < 0.0001).

**Fig. 4:**
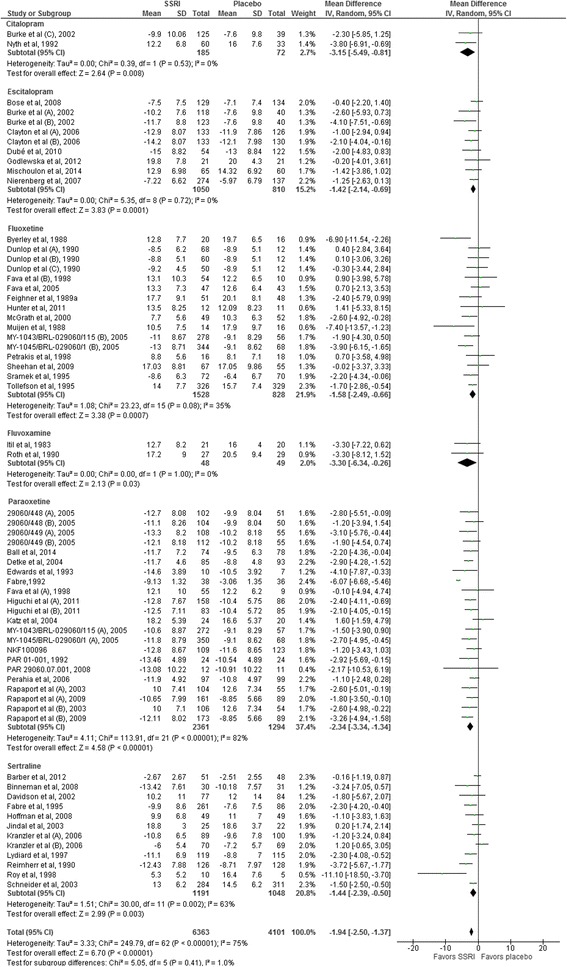
Subgroup analysis comparing trials assessing the effects of different selective serotonin reuptake inhibitors

**Fig. 5:**
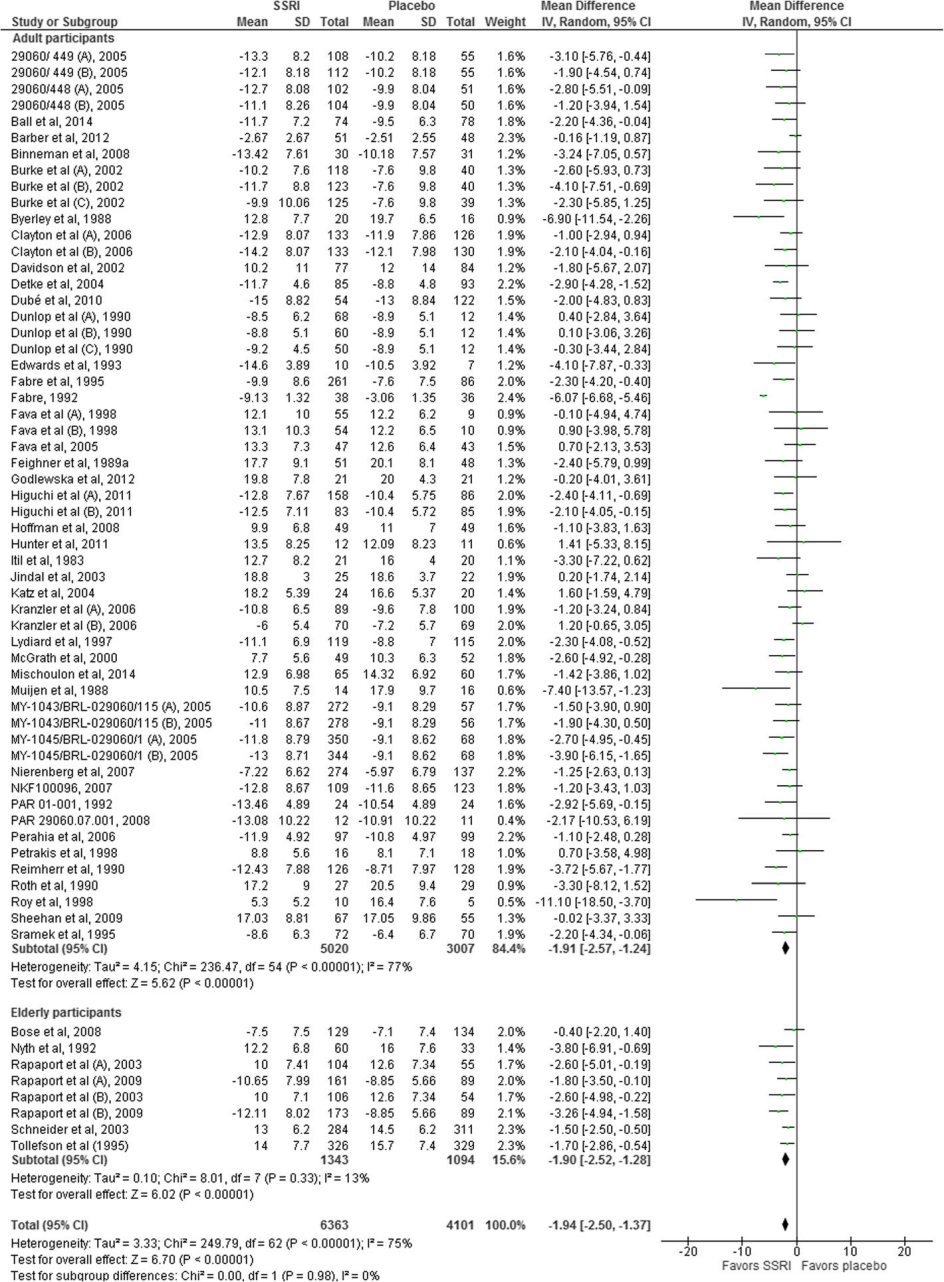
Subgroup analysis comparing trials randomising elderly participants to trials randomising non-elderly participants

**Fig. 6:**
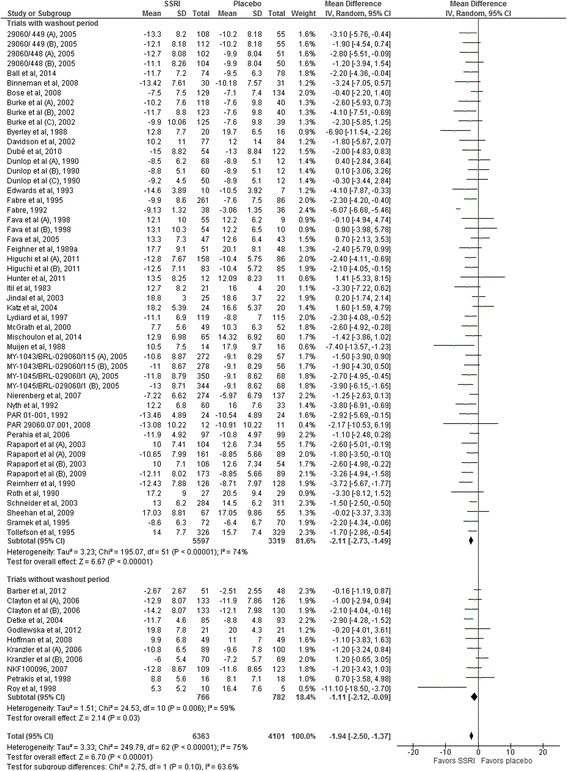
Subgroup analysis comparing trials using a placebo-washout period to trials not using a placebo-washout period

The following tests for subgroup differences did not show any significant differences: trials assessing the effects of the different SSRIs (number of trials 49; *I* ^2^ = 2.2%; *P* = 0.40) (Fig. 4); published trials (45 trials) compared to unpublished trials (4 trials): *I* ^2^ = 25.2%; *P* = 0.25; trials randomising elderly participants (6 trials) compared to younger participants (43 trials): *I* ^2^ = 0%; *P* = 0.94 (Fig. 5); trials with washout period (40 trials) compared to trials without washout period (9 trials): *I* ^2^ = 63.6%; *P* = 0.10 (Fig. 6); trials randomising drug or alcohol dependant participants (3 trials) compared to the remaining trials (46 trials): *I* ^2^ = 0%; *P* = 0.58; trials with an intervention period below 8 weeks (19 trials) compared to the remaining trials (30 trials): *I* ^2^ = 36.6; *P* = 0.21; and dose of the chosen SSRI (dose below the median (6 trials) compared to equal to or above the median (9 trials) of the SSRI: *I* ^2^ = 0%; *P* = 0.65. The latter two subgroup analyses were post-hoc analyses.

We performed an additional post-hoc subgroup analysis comparing trials with low risk of financial bias to trials with high risk of financial bias (Additional file 4: Figure S1). Test for subgroup differences showed no significant difference (*P* = 0.18). When the four trials with low risk of bias of financial bias were analysed separately then there was no significant difference between the SSRI group and the placebo group (−0.92 points; 95% CI −2.42 to 0.58; I^2^ 26%) (Additional file 4: Figure S1).

#### Trial Sequential Analysis

**Fig. 7:**
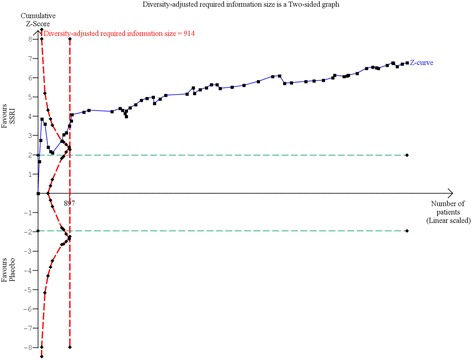
Trial Sequential Analysis of the results of selective serotonin reuptake inhibitors on Hamilton depression rating scale

The required information size was calculated based on an anticipated intervention effect of 3 HDRS points, the empirical variance, a risk of type I error of 0.025, and a power of 80% [12, 13, 17]. The Trial Sequential Analysis showed that the trial monitoring boundary for benefit was crossed after the 9th trial indicating a statistically significant result (Trial Sequential Analysis-adjusted CI −2.62 to −1.26) (Fig. 7).

#### Bayes factor

Bayes factor was calculated based on our anticipated intervention effect of 3 HDRS points and the primary meta-analysis result (mean difference −1.94 points; 95% CI −2.50 to −1.37) [12, 13, 17]. Bayes factor (2.01*10^−23^) was below the threshold for significance of 0.1, supporting the statistical significant result.

#### Risk of publication bias

**Fig. 8:**
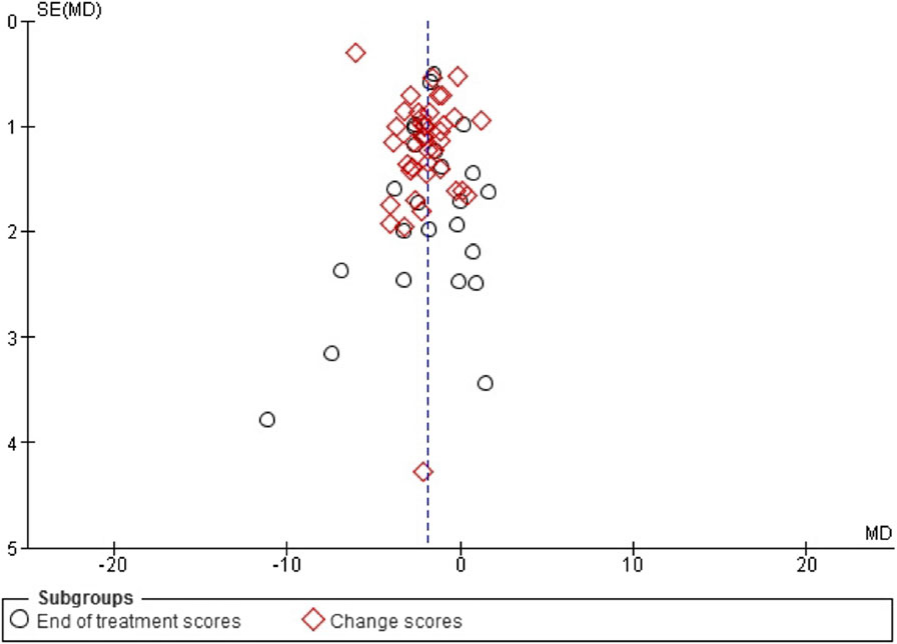
Funnel plot of the random-effects meta-analysis of the effect of selective serotonin reuptake inhibitors on Hamilton depression rating scale

Visual inspection of the funnel plot did not show clear signs of asymmetry (Fig. 8) [13, 17].

### Hamilton Depression Rating Scale, Montgomery-Asberg Depression Rating Scale, and Beck’s Depression Inventory

Sixty-one trials [32–52, 54–66, 103–106, 120, 121, 123, 137–142, 144–146, 149–151, 154, 155, 168, 172, 173] reported means and SD on the HDRS [18], MADRS [19], or BDI [174]. Using standardised mean difference, random-effects meta-analysis showed that SSRI versus placebo significantly decreased the standardised mean difference score (trials reporting mean scores: −0.23; 95% CI −0.31 to −0.14; *P* < 0.00001; trials reporting mean change scores: −0.26; 95% CI −0.35 to −0.17; *P* < 0.00001). The standardised mean difference was below our predefined threshold for clinical significance.

#### Long-term follow-up

One trial [33] reported mean HDRS scores and SD and one trial [146] reported mean HDRS change scores and SD at end of long-term follow-up. Random-effects meta-analysis of these trials showed a mean difference −0.18 points (95% CI −2.78 to 2.43; *P* = 0.89). Four trials reported mean HDRS scores or change scores at end of long-term follow-up but without reporting SDs [70, 97, 107, 143]. SDs were imputed. Random-effects meta-analysis of all the six trials showed a mean difference of −1.30 points (95% CI −2.72 to 0.13; *P* = 0.07).

#### No remission

**Fig. 9:**
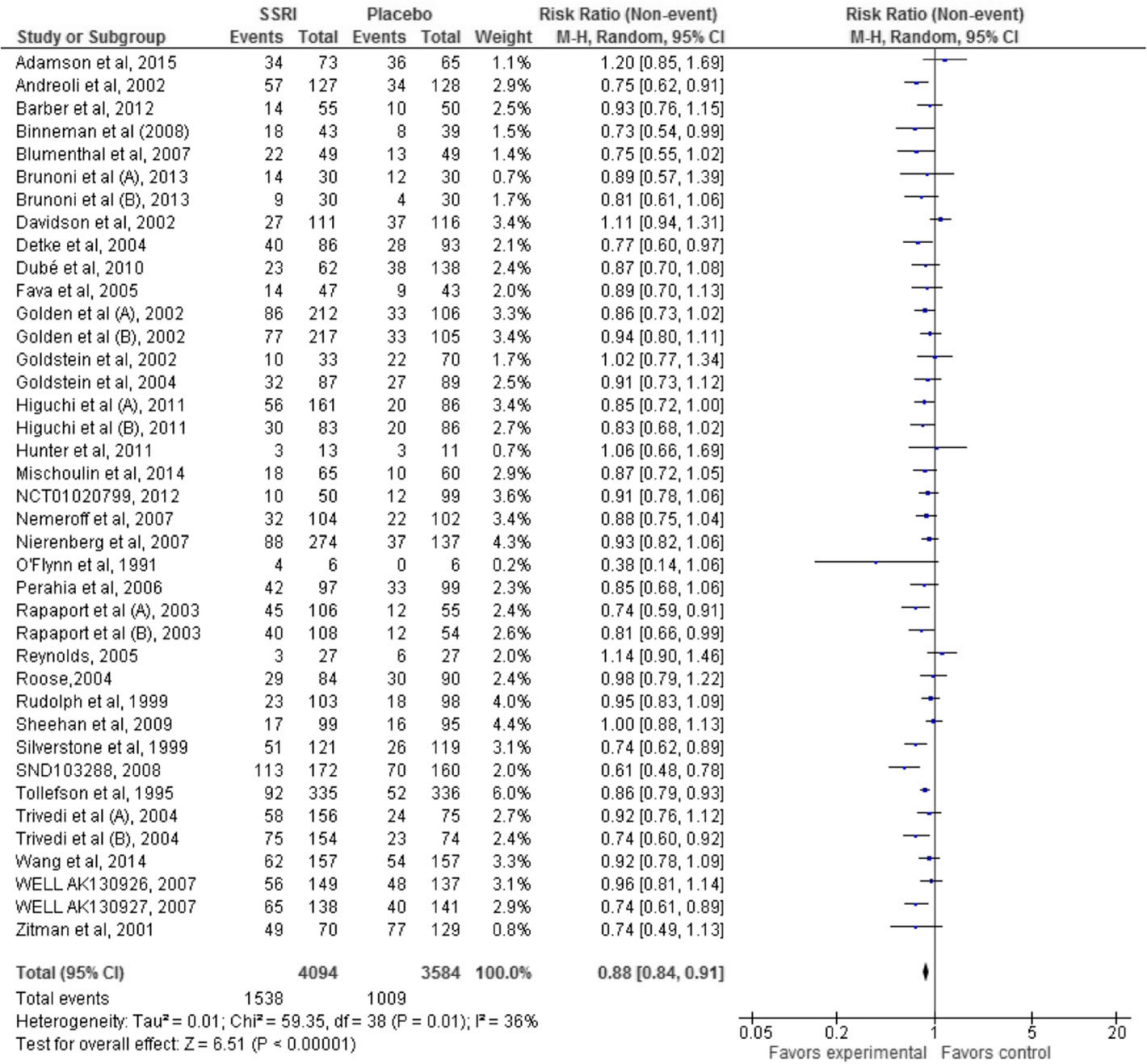
Random-effects meta-analysis of the results of selective serotonin reuptake inhibitors on remission of depression

Thirty-four trials [33, 34, 38, 45, 49–53, 55, 56, 60, 78, 81, 101, 104, 107–112, 120, 128, 140, 141, 146, 153, 157, 158, 162, 171, 175, 176] reported the proportion of participants with no remission at end of treatment. A total of 1430/2211 (64.7%) SSRI participants experienced no remission compared with 1493/2003 (74.5%) control participants. Random-effects meta-analysis showed that SSRIs versus placebo significantly decreased the risk of no remission (RR 0.88; 95% CI 0.84 to 0.91; *P* < 0.00001) (Fig. 9). This corresponds to 657 (95% CI 642 to 679) SSRI participants out of 1000 will experience no remission compared with 746 control participants out of 1000 (see Additional file 5: Summary of findings table). Visual inspection of the forest plots showed no clear signs of heterogeneity [13, 16].

**Fig. 10:**
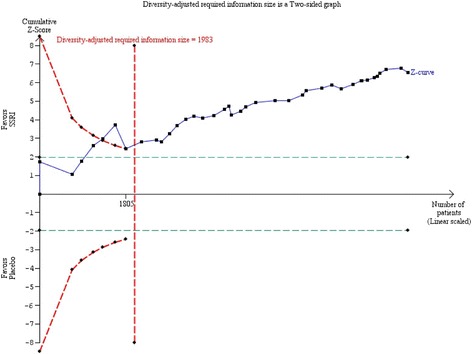
Trial Sequential Analysis of the results of selective serotonin reuptake inhibitors on remission of depression

The required information size was calculated based on the observed proportion of control participants without remission, a relative risk reduction of 30%, a risk of type I error of 0.025, and a power of 80% [13, 17]. The Trial Sequential Analysis showed that the trial monitoring boundary for benefit was crossed (Fig. 10) and the Trial Sequential Analysis-adjusted CI was 0.83 to 0.92 [13, 17].

Bayes factor was 1426.8 based on the random-effects meta-analysis result and above the threshold for significance of 0.1 [13, 17]. This relatively high Bayes factor indicates that it is more likely that null effect (null hypothesis) compared to the anticipated intervention effect has produced this meta-analysis results [12, 13].

Best-worst case scenario showed a highly significant meta-analysis result (RR 0.78; 95% CI 0.73 to 0.83; *P* < 0.00001) [13, 17]. Worst-best case scenario showed no significant difference on risk of no remission (RR 0.95; 95% CI 0.89 to 1.02; *P* = 0.14) [13, 17].

Visual inspection of the funnel plot showed no clear signs of publication bias [13, 16, 17].

#### Serious adverse events

**Fig. 11:**
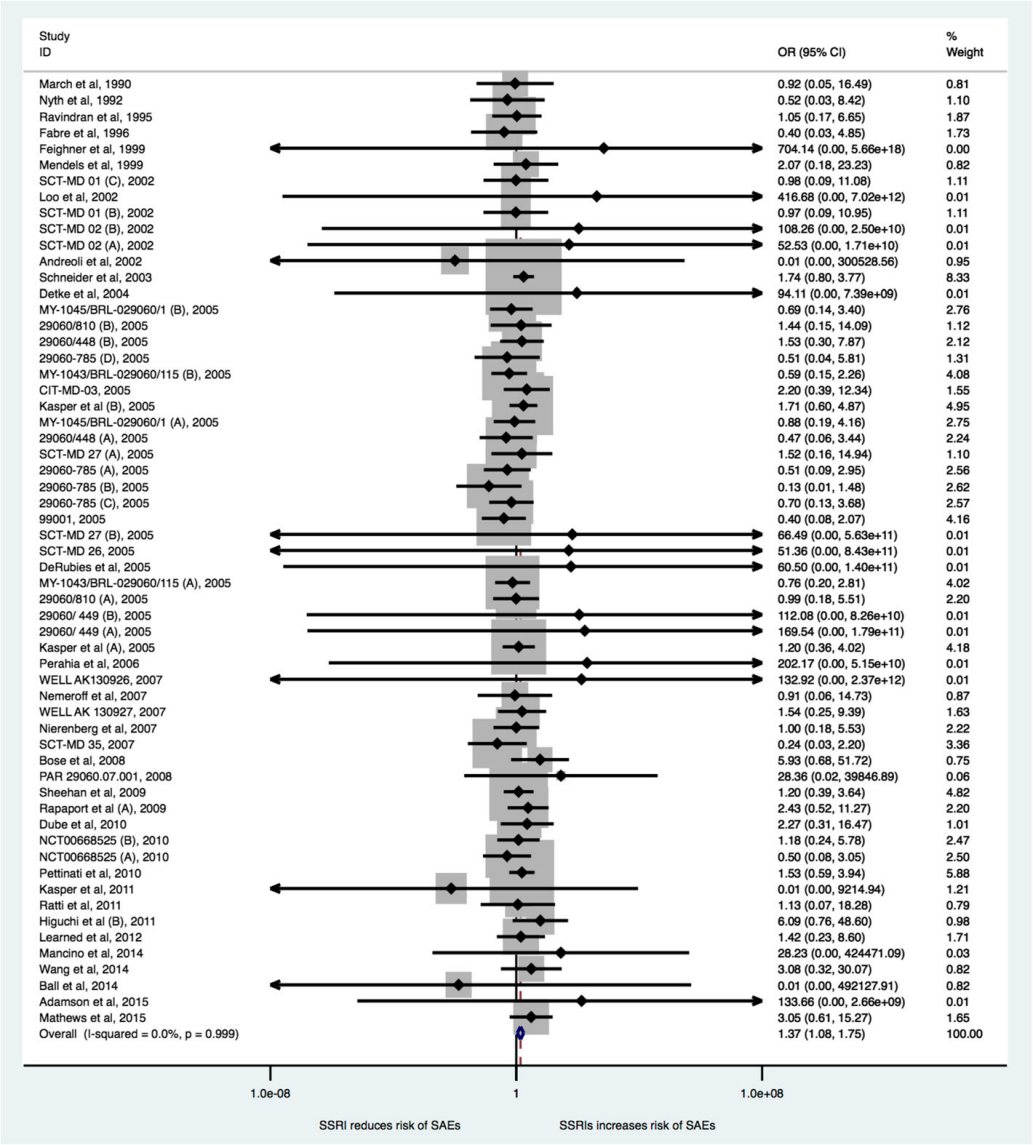
Random-effects meta-analysis of the results of selective serotonin reuptake inhibitors on serious adverse events

**Fig. 12:**
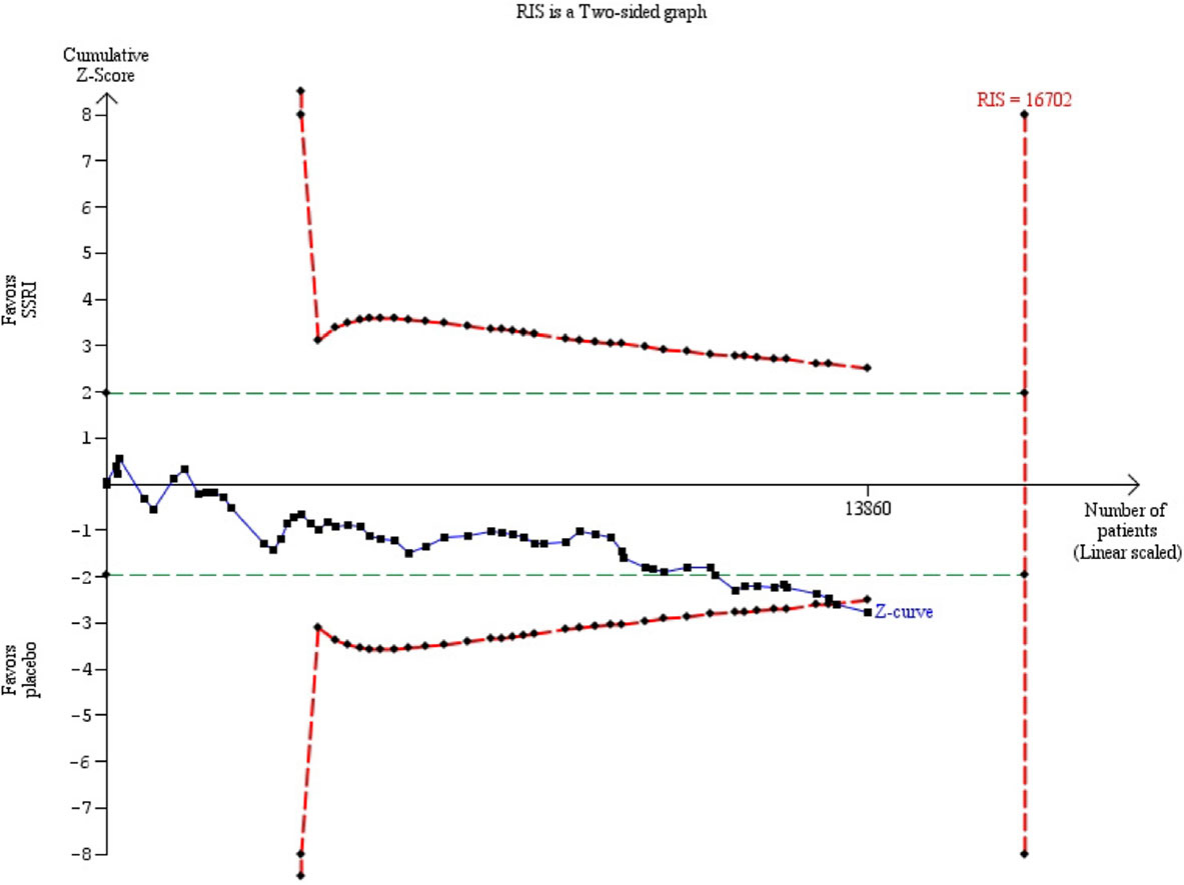
Trial Sequential Analysis of the results of selective serotonin reuptake inhibitors on serious adverse events

Because of the low proportion in the control group (around 2%) we used the Mantel–Haenszel (MH) odds ratio method with reciprocal zero cell correction (zero is replaced by the reciprocal of the size of the opposite treatment arm) [177]. Forty-four trials reported the proportion of participants with serious adverse events [48, 49, 54–56, 60, 63, 75, 78, 93, 94, 102, 105, 108, 112–118, 120, 121, 137–139, 141, 144–146, 149–151, 155, 167, 171, 173, 176, 178–180]. A total of 239/8242 (2.7%) SSRI participants experienced a serious adverse event compared with 106/4956 (2.1%) control participants. Random-effects meta-analysis showed that SSRIs versus placebo significantly increased the risk of a serious adverse event (OR 1.37; 95% CI 1.08 to 1.75; *P* = 0.009) (Fig. 11). This corresponds to 31 (95% CI 25 to 40)/1000 SSRI participants will experience a serious adverse event compared with 22/1000 control participants (see Additional file 5: Summary of findings table). Visual inspection of the forest plot did not indicate significant heterogeneity [13, 17]. Even when using the multiplicity adjusted risk of type I error (0.05/2 = 0.025), the trial sequential boundary for harm was crossed (Fig. 12) and the Trial Sequential Analysis-adjusted CI was 1.03 to 1.89 [13, 17]. Table 2 summarises the types of adverse events.

**Table 2:** Summary of serious adverse events in the included trials

Trial	Experimental intervention	SSRI participants assessed for serious adverse events		Placebo participants assessed for serious adverse events	
		Numbers and types of serious adverse events	Proportion of participants with a serious adverse event	Numbers and types of serious adverse events	Proportion of participants with a serious adverse event
Bose et al., 2008	Escitalopram	1 bowel obstruction, 1 nausea, 1 arrythmia, 1 respiratory arrest, 1 retinal detachment, 1 chest pain	5 out of 96	1 syncope	1 out of 109
Ball et al., 2014	Paroxetine	No serious adverse event	0 out of 74	1 unspecified serious adverse event	1 out of 78
Andreoli et al., 2002	Fluoxetine	No serious adverse event	0 out of 97	1 suicide	1 out of 76
Kasper et al., 2011	Escitalopram	No serious adverse event	0 out of 128	1 hospitalisation due to appendicitis	1 out of 50
Kasper et al. (A), 2005	Escitalopram	1 death (suicide)	1 out of 173	1 death (probably drowned)	1 out of 160
Ravindran et al., 1995	Sertraline	9 unspecified serious adverse events	4 out of 25	3 unspecified serious adverse events	2 out of 13
March et al., 1990	Fluvoxamine	1 hospitalisation due to worsening of depression	1 out of 13	1 suicide attempt	1 out of 12
Rapaport et al., 2009	Paroxetine	2 chestpain, 1 osteoarthritis, 1 ankle fracture, 1 atrial fibrillation, 1 femur fracture,1 coronary artery occlusion, 1 pneumonia, 1 confusional state, 1 depression	10 out of 267	1 neprholithiasis, 1 aortic aneurism	2 out of 127
Higuchi et al., 2011	Paroxetine	1 suicide and 8 unspecified serious adverse events	9 out of 213	1 unspecified serious adverse event	1 out of 139
Schneider et al., 2003	Sertraline	17 unspecified serious adverse events	17 out of 284	11 unspecified serious adverse events	11 out of 311
Sheehan et al., 2009	Fluoxetine	1 suicidal ideation/suicidality, 1 worsening of depression, 2 suicide attempts, 1 anxiety/agitation/racing thoughts, 1 syncope, 1 ankle fracture, 1 viral gastro enteritis	8 out of 76	2 suicidal ideation/suicidality, 2 worsening of depression, 1 nose bleed, 1 allergic reaction	6 out of 67
Nemroff et al., 2005	Fluxetine	1 unspecified serious adverese event	1 out of 86	1 unspecified serious adverse event	1 out of 78
Fabre et al., 1996	Fluvoxamine	1 hospitalisation (non-cardiac chest pain)	1 out of 22	1 hospitalisation, 1 ruptured ectopic pregnancy, 1 hernia repair	2 out of 19
Feighner et al., 1999	Citalopram	3 suicide attempts, 1 miscarriage, 1 intestinal flu symptoms, 1 chest pain, 1 severe thinking abnormality, 1 allergic reaction	8 out of 349	No serious adverse event	0 out of 86
SCT-MD 01 (B), 2002	Escitalopram	1 anaphylaxis, 1 suicide attempt	2 out of 94	1 gallbladder stones	1 out of 46
SCT-MD 01 (C), 2002	Citalopram	1 coma, 1 intestinal fistula	2 out of 93	1 non-accidental overdose	1 out of 45
SCT-MD 02 (A), 2002	Escitalopram	1 suicidal tendency, suicide attempt; 1 non-accidental overdose, suidal attempt, tachycardia	2 out of 96	No serious adverse event	0 out of 53
SCT-MD 02 (B), 2002	Citalopram	1 cholestasis intrahepatic, dehydration	1 out of 99	No serious adverse event	0 out of 52
Pettinati et al., 2010	Sertraline	15 unspecified serious adverse events	15 out of 40	11 unspecified serious adverse events	11 out of 39
Dube et al., 2010	Escitalopram	1 suicide attempt, 1 gastro enteritis/malaria	2 out of 54	1 near drowning, 1 gastro enteritis	2 out of 122
Learned et al., 2012	Paroxetine	1 intentional over dose, 1 depression, 1 unspecified event	3 out of 166	2 unspecified serious adverese events	2 out of 156
Ratti et al., 2011	Paroxetine	1 hemorrhoidal hemorrhage	1 out of 109	1 rash	1 out of 123
Wang et al., 2014	Escitalopram	3 unspecified serious adverese event	3 out of 114	1 unspecified serious adverese event	1 out of 115
Detke et al., 2004	Paroxetine	1 unspecified serious adverese event	1 out of 85	No serious adverse event	0 out of 93
Mancino et al., 2014	Sertraline	1 hospitalization	1 out of 23	No serious adverse event	0 out of 27
DeRubeis et al., 2005	Paroxetine	1 suicide	1 out of 120	No serious adverse event	0 out of 60
29060-785 (A), 2005	Paroxetine CR 25 mg	3 abnormal laboratory vaue, 1 emotional lability	4 out of 98	1 abnormal laboratory value, 1 gastrointestinal disorder	2 out of 26
29060-785 (B), 2005	Paroxetine CR 12.5 mg	1 abnormal laboratory value	1 out of 94	1 abnormal laboratory value, 1 myocardial infarction	2 out of 26
29060-785 (C), 2005	Citalopram 20 mg	5 abnormal laboratory value, 1 syncope	6 out of 105	1 abnormal laboratory value, 1 suicide	2 out of 25
29060-785 (D), 2005	Citalopram 40 mg	1 abnormal laboratory vaue, 1 emotional lability	2 out of 97	1 abnormal laboratory value	1 out of 25
SCT-MD 27 (A), 2005	Escitalopram	1 depression, 1 abnormal mental status, 1 malignant neoplasm	3 out of 131	1 labyrinthitis	1 out of 66
SCT-MD 27 (B), 2005	Sertraline	1 appendicitis	1 out of 135	No serious adverse event	0 out of 66
SCT-MD 35, 2007	Escitalopram	1 abnormal hepatic function	1 out of 131	1 breast cancer, 1 depression, 1 suicidal ideation, 1 suicide	4 out of 130
SCT-MD 26, 2005	Escitalopram	1 inflicted injury	1 out of 143	No serious adverse event	0 out of 151
MY-1043/BRL-029060/115 (A), 2005	Paroxetine	1 hypertension, 1 diabetes and hypothyroidism, 1 Fibrocystic disease, 1 Ovarian cysts, 1 peptic ulcer hemorrhage, 1 spinal surgery, 1 hypomanic episode with suicidal tendency, 2 Suicidal ideation, 1 alcoholism, 1 neoplasm	11 out of 272	1 suicidal ideation, 1 back pain, 1 trauma	3 out of 57
MY-1043/BRL-029060/115 (B)	Fluoxetine	1 suicidal ideation, 1 neoplasm, 2 acute pyelonephritis, 1 thrombophlebitis, 1 ectopic pregnancy, 1 polycystic granuloma, 2 basal cell carcinomas, 1 myxoid mitral valve	9 out of 278 (10 SAE in 9 participants)	1 viral meningitis, 1 infection, 1 myocardial infarction, 1 mole removal	3 out of 56 (4 SAE in 3 participants)
MY-1045/BRL-029060/1, (A), 2005	Paroxetine	2 depression (worsening), 2 emotional lability, 1 neoplasm, 1 insomnia, 1 nervousness, 1 carcinoma, 1 epistaxis, 1 gastro intestinal disorder, 1 prostate disorder	9 out of 357 (11 SAE in 9 participants)	1 depression (worsening), 1 rectal disorder	2 out of 70
MY-1045/BRL-029060/1, 2005	Fluoxetine	1 depression (worsening), 2 emotional lability, 1 neo plasm, 1 coronary artery disease, 1 thrombo phlebitis, 1 hypoglycemia	7 out of 351	2 depression (worsening), 1 flu syndrome disorder	2 out of 70
29060/448 (A), 2005	Paroxetine IR	1 myocardial infarction, 1 emotional lability	2 out of 104	1 uterine fibroids enlarged, 1 gall bladder disorder	2 out of 50
29060/448 (B), 2005	Paroxetine CR	3 emotional lability, 1 hepatocellular jaundice, 1 manic reaction	6 out of 102	1 dehydration, 1 accidental overdose	2 out of 51
29060/449 (A), 2005	Paroxetine IR	1 emotional lability, 1 abortion, 2 unintended pregnancy	3 out of 112 (4 SAE in 3 participants)	No serious adverse event	0 out of 55
29060/449 (B), 2005	Paroxetine CR	1 abdominal pain, 1 pancreatitis, 1 accidental over dose, 1 unintended pregnancy	2 out of 108 (4 SAE in 2 participants)	No serious adverse event	0 out of 55
PAR 29060.07.001, 2008	Paroxetine	1 acure depression, 1 acute alcohol intoxication and suicide ideation	2 out of 13	No serious adverse event	0 out of 12
Nyth et al., 1992	Citalopram	1 cerebral hemorrhage and death	1 out of 98	1 death	1 out of 51
Perahia et al., 2006	Paroxetine	1 back pain, 1 breast neoplasm	2 out of 97	No serious adverse events	0 out of 99
Mendels et al., 1999	Citalopram	1 prostatic hyper trophy, 1 bronchitis	2 out of 89	1 suicide	1 out of 91
NCT00668525 (A), 2010	Escitalopram	1 chest pain, 1 pharyngitis 1 multiple sclerosis	3 out of 319	1 asthma, 1 haemothorax	2 out of 108
NCT00668525 (B), 2010	Escitalopram	1 chest pain, 1 appendicitis, 2 anxiety, 1 suicidal ideation, 1 suicide attempt, 1 peripheral vasuclar disorder	7 out of 318	1 injury, 1 suicidal ideation	2 out of 107
Nierenberg et al., 2007	Escitalopram	1 death and 3 unspecified serious adverse events	4 out of 274	2 unspecified serious adverse events	2 out of 137
WELL AK130926, 2007	Escitalopram	1 agitation	1 out of 144	No serious adverse event	0 out of 132
WELL AK130927, 2007	Escitalopram	2 suicidal ideation, 1 hepatic function abnormal	3 out of 138	1 suicidal ideation, 1 sudden caridac death	2 out of 141
NCT01473381, 2014	Escitalopram	1 haemorrhagic anaemia, 1 diverticulitis, 1 ilium fracture, 1 road traffic accident, 1 traumatic renal injury, 1 wrist fracture, 1 abortion missed, 1 suicidal ideation, 1 hospitalisaation	6 out of 280 (9 SAE in 6 participants)	1 angina pectoris, 1 gastric disorder, 1 pneumonia, 1 neck abscess, 1 oral abscess, 1 abnormal electrocardiogram ST segment, 1 back pain, 1 suicidal ideation, 1 obstructive airways disorder	3 out of 281 (9 SAE in 3 participants)
Adamson et al., 2015	Citalopram	1 suicidal ideation, severe abdominal cramps	2 out of 73	No serious adverse event	0 out of 65
CIT-MD-03, 2005	Citalopram	2 congestive heart failure, 1 cerebro vascular accident, 1 hyponatremia	4 out of 84	1 cerebro vascular accident, 1 cellulitis	2 out of 90
29060/810 (A), 2005	Paroxetine CR 12.5 mg	2 abnormal laboratory value, 1 carcinoma of lung	3 out of 153	1 cerebro vascular disorder, 1 depression	1 out of 73 (2 SAE in 1 participants)
29060/810 (B), 2005	Paroxetine CR 25 mg	1 abnormal laboratory value, 1 gall bladder disorder, 1 anxiety, 1 emotional lability,	4 out of 148	1 pleura disorder, 1 sinusitis, 1 bronchitis	2 out of 73 (3 SAE in 2 participants)

*SAE* Serious adverse event

Bayes factor was 4.8*10^5^ above the threshold for significance of 0.1 [13, 17]. This clearly shows that a beneficial effect of SSRIs on serious adverse events is very unlikely [13, 17]. Visual inspection of the funnel plot showed no signs of publication bias [13, 16, 17]. Based on the random-effects meta-analysis result, we calculated the number-needed-to-seriously harm one patient to be 138 patients.

#### Adverse events

Meta-analyses showed that the participants randomised to SSRIs versus placebo had a significantly increased risk of several adverse events. We have summarised the risks of the adverse events which were most reported in Additional file 6, including numbers-needed-to-harm. We have also included forest plots for the 25 most statistically significant adverse event results in the Additional files (see Additional files 7, 8, 9, 10, 11, 12, 13, 14, 15, 16, 17, 18, 19, 20, 21, 22, 23, 24, 25, 26, 27, 28, 29, 30 and 31: Figure S3-S27). The full list of the 84 reported adverse events are summarised in Table 3.

**Table 3:** Summary of all reported adverse events in the included trials

Event	No. of trials reporting the event	SSRI		Placebo		Relative risk (95% CI)	Number needed to harm (NNH)	P value
		Number of participants with the event	Number of participants randomised	Number of participants with the event	Number of participants randomised			
Abnormal ejaculation	15	183	3236	7	1903	5.43 [3.22, 9.14]	19	*P* < 0.00001
Tremor	28	301	3502	61	2929	3.16 [2.37, 4.21]	16	*P* < 0.00001
Anorexia	19	220	2350	42	1680	2.78 [2.03, 3.79]	15	*P* < 0.00001
Nausea	78	2524	12,257	779	8491	2.48 [2.22, 2.77]	9	*P* < 0.00001
Somnolence	59	1336	10,351	345	6674	2.25 [2.00, 2.53]	13	*P* < 0.00001
Sweating	34	440	5274	124	3478	2.20 [1.80, 2.70]	21	*P* < 0.00001
Asthenia	23	497	3968	155	2265	1.71 [1.43, 2.04]	18	*P* < 0.00001
Diarrhoea	58	1458	11,056	561	7099	1.66 [1.51, 1.83]	19	*P* < 0.00001
Constipation	50	606	6698	273	4892	1.60 [1.35, 1.89]	29	*P* < 0.00001
Insomnia	69	1500	11,934	582	7956	1.49 [1.35, 1.64]	19	*P* < 0.00001
Dizziness	55	849	8900	398	6161	1.39 [1.24, 1.57]	33	*P* < 0.00001
Dry mouth	73	1376	11,303	693	7904	1.37 [1.25, 1.49]	30	*P* < 0.00001
Libido decreased	8	78	1481	11	1083	3.48 [1.92, 6.32]	24	*P* < 0.0001
Sexual dysfunction	6	96	719	16	389	2.85 [1.77, 4.59]	11	*P* = 0.0001
Appetite decreased	8	68	932	24	885	2.63 [1.66, 4.17]	22	*P* < 0.0001
Fatigue	26	409	5098	153	3545	1.69 [1.32, 2.17]	27	*P* < 0.0001
Vomiting or upset stomach	20	189	2376	101	2037	1.55 [1.16, 2.08]	34	*P* = 0.003
Flu syndrome	7	57	1069	19	822	2.13 [1.28, 3.54]	34	*P* = 0.004
Drowsiness	5	38	253	19	256	1.90 [1.18, 3.04]	14	*P* = 0.004
Blurred/abnormal vision or dry eyes	17	116	1862	55	1566	1.55 [1.15, 2.10]	37	*P* = 0.004
Nervousness	22	484	3863	147	2043	1.35 [1.10, 1.66]	19	*P* = 0.004
Back pain	11	85	2404	71	1594	0.66 [0.48, 0.91]	109	*P* = 0.01
Headache	72	2386	11,085	1427	7805	1.08 [1.01, 1.14]	31	*P* = 0.02
Dyspepsia	23	331	4304	159	2956	1.29 [1.04, 1.59]	44	*P* = 0.02
Weight loss	3	26	562	9	560	2.48 [1.17, 5.25]	34	*P* = 0.02
Hypertension	4	17	933	25	761	0.51 [0.28, 0.93]	69	*P* = 0.03
Central or peripheral nervous system	4	104	221	16	57	1.58 [1.03, 2.43]	6	*P* = 0.04
Lightedness/faint feeling	3	9	147	1	144	4.81 [1.06, 21.72]	19	*P* = 0.04
Accidental injury	3	15	672	23	516	0.50 [0.25, 0.99]	45	*P* = 0.05
Agitation	5	67	613	22	398	1.60 [1.01, 2.54]	19	*P* = 0.05
Impotence	3	19	868	1	603	3.12 [0.99, 9.88]	50	*P* = 0.05
Taste perversion	2	9	389	1	390	5.80 [1.02, 33.03]	49	*P* = 0.05
Shaking	2	7	121	0	116	7.19 [0.91, 57.03]	18	*P* = 0.06
Rhinitis	14	197	3004	171	1969	0.78 [0.63, 0.97]	47	*P* = 0.06
Palpitations	10	63	1572	30	1292	1.55 [0.97, 2.50]	60	*P* = 0.07
Infection	7	104	997	57	612	1.31 [0.97, 1.75]	90	*P* = 0.08
Amnesia	2	5	484	12	486	0.44 [0.16, 1.20]	70	*P* = 0.11
Psychiatric adverse effects	1	19	40	7	26	1.76 [0.87, 3.60]	5	*P* = 0.12
Sleep distrurbance	3	15	240	7	223	1.98 [0.83, 4.73]	33	*P* = 0.13
Sinusitis	4	35	751	38	629	0.69 [0.43, 1.11]	73	*P* = 0.13
Urinary frequency	4	40	695	19	624	1.80 [0.82, 3.96]	37	*P* = 0.13
Anxiety	17	150	2983	89	2250	1.27 [0.91, 1.77]	94	*P* = 0.15
Appetie increased	3	5	335	10	330	0.49 [0.17, 1.43]	65	*P* = 0.19
Coughing	1	8	100	4	96	1.92 [0.60, 6.17]	27	*P* = 0.27
Tinnitus	2	4	319	1	319	2.84 [0.44, 18.44]	107	*P* = 0.27
Adverse events overall	4	32	232	14	160	1.36 [0.77, 2.39]	20	*P* = 0.29
Unpleasant taste	1	2	21	0	20	4.77 [0.24, 93.67]	11	*P* = 0.30
Congestive heart failure	1	0	335	2	336	0.20 [0.01, 4.16]	168	*P* = 0.30
Gastrointestial	4	66	194	14	91	1.53 [0.65, 3.62]	6	*P* = 0.33
Autonomic nervous system	1	10	40	4	26	1.63 [0.57, 4.64]	11	*P* = 0.36
Respiratory disorder	10	244	2764	107	1276	0.90 [0.73, 1.12]	227	*P* = 0.36
Vasodialtion	4	11	368	4	355	1.75 [0.44, 6.94]	54	*P* = 0.43
Flatulence	6	94	1763	34	972	1.24 [0.72, 2.16]	55	*P* = 0.44
Malaise	1	0	21	1	20	0.32 [0.01, 7.38]	20	*P* = 0.48
Depression aggrevated	1	6	337	1	180	1.95 [0.31, 12.35]	82	*P* = 0.48
Female genital disorders	1	7	310	1	149	1.93 [0.31, 12.10]	63	*P* = 0.48
Weight gain	1	3	129	5	129	0.60 [0.15, 2.46]	65	*P* = 0.48
Tachycardia	6	9	989	6	996	1.43 [0.52, 3.96]	326	*P* = 0.49
Arrhythmia	1	0	335	1	336	0.33 [0.01, 8.18]	336	*P* = 0.50
Atrial fibrillation	1	1	335	0	336	3.01 [0.12, 73.60]	335	*P* = 0.50
Abnormal electrocardiogram	1	1	335	0	336	3.01 [0.12, 73.60]	335	*P* = 0.50
Migraine	1	1	129	0	129	3.00 [0.12, 72.96]	129	*P* = 0.50
Chest discomfort	1	1	21	0	20	2.86 [0.12, 66.44]	21	*P* = 0.51
Rash	5	9	280	12	282	0.79 [0.37, 1.70]	97	*P* = 0.55
Vertigo	1	10	337	3	180	1.58 [0.32, 7.68]	77	*P* = 0.57
Dysuria	1	2	21	1	20	1.90 [0.19, 19.40]	23	*P* = 0.58
Pruritus (Itching)	3	8	187	10	185	0.79 [0.30, 2.10]	89	*P* = 0.63
Orthostatic hypotension	2	6	466	3	309	1.37 [0.37, 5.02]	316	*P* = 0.64
Upper respiratory tract infection	14	164	2380	123	1882	0.95 [0.75, 1.20]	282	*P* = 0.68
Body as a whole	1	6	40	3	26	1.30 [0.36, 4.75]	29	*P* = 0.69
Loose stools	1	3	100	2	96	1.44 [0.25, 8.43]	110	*P* = 0.69
Gastritis	1	3	62	5	138	1.34 [0.33, 5.41]	83	*P* = 0.69
Cardiovascular disorder	2	10	444	3	355	0.81 [0.22, 2.95]	72	*P* = 0.75
Pain	5	65	893	59	729	0.95 [0.68, 1.34]	123	*P* = 0.77
Abnormal thinking	1	7	335	8	336	0.88 [0.32, 2.39]	344	*P* = 0.80
Abnormal acne	1	3	32	1	14	1.31 [0.15, 11.54]	45	*P* = 0.81
Confusion	4	5	640	5	621	0.87 [0.28, 2.74]	4187	*P* = 0.81
Myalgia	5	34	692	26	572	0.93 [0.45, 1.94]	272	*P* = 0.85
Irritability	6	18	643	20	642	0.92 [0.38, 2.27]	317	*P* = 0.86
Numbness	2	12	272	8	269	0.77 [0.04, 13.27]	70	*P* = 0.86
Abdominal pain	9	98	1967	52	1150	1.02 [0.73, 1.44]	218	*P* = 0.89
Trauma	2	47	883	14	267	0.95 [0.41, 2.20]	1261	*P* = 0.90
Eructation (burping)	1	5	100	5	96	0.96 [0.29, 3.21]	480	*P* = 0.95
Over sedation	3	24	677	7	514	1.91 [0.83, 4.39]	46	*P* =0.13

#### Clinical significance

All primary HDRS meta-analyses showed intervention effect estimates below our predefined threshold for clinical significance (a mean difference of 3 HDRS points or 0.5 standardised mean difference) [13, 17]. Our results show statistically significant effects, but the possible effects all seem to have questionable clinical significance [13].

### Secondary outcomes

#### Suicides, suicide attempts, and suicide ideation

There were no significant differences between participants randomised to SSRIs versus placebo on number of suicides (RR 0.68; 95% CI 0.16 to 2.81; *P* = 0.59; Trial Sequential Analysis-adjusted CI 0.01 to 226.85; 6 trials [60, 71, 108, 113, 151, 155]); suicide attempts (RR 1.76; 95% CI 0.59 to 5.22; *P* = 0.31; Trial Sequential Analysis-adjusted CI 0.02 to 149.95; 8 trials [49, 56, 75, 94, 102, 139, 167, 181]); or suicide ideation (RR 0.80; 95% CI 0.36 to 1.77; *P* = 0.58; Trial Sequential Analysis-adjusted CI 0.03 to 23.20; 11 trials [49, 51, 120, 138, 139, 145, 151, 162, 167, 171, 180]). The required information size was not reached in any of the three Trial Sequential Analyses.

#### Quality of life

Only six trials assessed quality of life [48, 51, 63, 100, 101, 112] out of which four trials reported results on the quality of life enjoyment and satisfaction questionnaire (Q-LES-Q) [48, 51, 100, 101]. Two trials [48, 101] reported mean scores and SDs. Random-effects meta-analysis showed significant effect of SSRI on Q-LES-Q scores (RR 2.98; 95% CI 1.34 to 4.61; *P* = 0.0004).

Two trials reported results on the short form of the quality of life enjoyment and satisfaction questionnaire [63, 112], but only one trial reported mean scores and SDs [63]. The results from this trial showed that SSRIs (paroxetine) versus placebo significantly increased the mean score of the questionnaire (paroxetine 12.5 mg group mean 11.4, SD 16.7; paroxetine 25 mg group mean 11.5, SD 17.2; placebo group mean 5.3, SD 17.1) [63].

#### Post hoc analysis of no response

We identified 70 trials assessing the effects of SSRIs on no response defined as less than 50% reduction (from baseline) on either HDRS or MADRS. The meta-analysis showed that SSRIs seem to significantly decrease the risk of no response compared with placebo (RR 0.83; 95% CI 0.80 to 0.87; *P* = 0.00001) (Additional file 32: Figure S2).

#### GRADE assessments

GRADE assessments show that due to the high risks of bias the quality of the evidence must be regarded as very low (Additional file 5: Summary of Findings Table) [13].

## Discussion

SSRIs may affect the concentration of essential neurotransmitter substances in the brain and are therefore considered to exert effects on depressive symptoms. However, whether these effects are beneficial and clinically meaningful are the questions. Estimating a meaningful threshold for clinical significance is difficult and an assessment of clinical significance should ideally not only include a threshold on an assessment scale [182]. Major depressive disorder affects daily functioning, increases the risk of suicidal behaviour, and decreases quality of life [183]. Some adverse events might therefore be acceptable if SSRIs have clinically significant beneficial effects [13, 183, 184]. We therefore both predefined a threshold for clinical significance and assessed the balance between beneficial and harmful effects [13, 17, 184].

As threshold for clinical significance [14], we chose a drug-placebo difference of 3 points on the 17-item HDRS (ranging from 0 to 52 points) or an effect size of 0.50 standardised mean difference. This has been recommended by the National Institute for Clinical Excellence (NICE) in England and has been chosen in other reviews [4, 8, 31]. Nevertheless, these recommendations are not universally accepted and have been questioned [3]. Others have suggested the following ‘rules of thumb’ regarding the standardised mean difference: 0.2 a small effect, 0.5 a moderate effect, and 0.8 a large effect [16, 185]. One study has shown that a SSRI-placebo mean difference of up to three points on the HDRS corresponds to ‘no clinical change’ [186]. Another valid study has shown that a SSRI-placebo difference of 3 points is undetectable by clinicians, and that a mean difference of 7 HDRS points, or a standardized mean effect size of 0.875, is required to correspond to a rating of ‘minimal improvement’ [187]. It has been speculated that the ‘placebo’ response in antidepressant trials has been increasing during recent years [188]. If there is a ‘response’ to placebo this has of course to be considered when interpreting a mean difference between drug and placebo. However, it is unlikely that depressed patient have a significant placebo effect [189] and it has recently been shown that the placebo response has been stable for 25 years [188]. Even based on our predefined minimal thresholds for clinical significance, the effects of SSRIs did not have a clinically meaningful effect on depressive symptoms. Furthermore, per our meta-analyses SSRIs significantly increase the risk of both serious and non-serious adverse events.

The best-worst and worst-best case scenarios showed that incomplete outcome data bias alone theoretically could have caused the apparent statistically significant beneficial effect of SSRIs. Furthermore, seen in the light of the total number of trials, only a relatively limited number of trials reported on each of our pre-defined outcomes. This increases the risk of selective outcome reporting bias. Apart from the high risk of incomplete outcome data bias and selective outcome reporting bias, all the included trials were assessed at high risk of bias. All trials used placebo as control intervention and due to the large number of adverse events, some patients might have figured out if they received an ‘active’ intervention or not, which might question the blinding of the trials. Nevertheless, it may be argued that our bias risk assessment often will lead to no trials with low risk of bias. However, similar bias risk assessments have been used in several previous systematic review (see, e.g., most Cochrane Hepato-Biliary Group systematic reviews) and our bias risk assessment is based on valid evidence clearly showing that if each of the used bias risk domains is ‘high risk of bias’ or ‘unclear risk of bias’ then there is a risk of overestimation of benefits and underestimation of harms [184, 190–197]. Furthermore, the risks of bias observed here just mirrors our experience in 786 randomised trials on depression [198].

We chose ‘remission’ as a primary outcome because we expected trialists to use this outcome frequently. To present a complete overview of the evidence on SSRIs for depression we also included ‘no response’ (less than 50% reduction on HDRS or MADRS during the intervention period) in a post hoc analysis because this outcome was frequently used in the included trials and by requests from peer reviewers. However, our results on no remission and no response should be interpreted with great caution for a number of reasons: 1) the assessments of remission and response were primarily based on single HDRS scores and it is questionable whether single HDRS scores are indications of full remission or adequate response to the intervention; 2) information is lost when continuous data are transformed to dichotomous data and the analysis results can be greatly influenced by the distribution of data and the choice of an arbitrary cut-point [16, 199–201]; 3) even though a larger proportion of participants cross the arbitrary cut-point in the SSRI group compared with the control group (often HDRS below 8 for remission and 50% HDRS reduction for response), the effect measured on HDRS might still be limited to a few HDRS points (e.g., 3 HDRS points) or less; 4) by only focusing on how many patients cross a certain line for benefit, investigators ignore how many patients are deteriorating at the same time. If results, e.g., show relatively large beneficial effects of SSRIs when remission and response are assessed but very small averaged effects (as our results show) – then it must be because similar proportions of the participants are harmed (increase on the HDRS compared to placebo) by SSRIs. Otherwise the averaged effect would not show small or no difference in effect. The clinical significance of our results on ‘no remission’ and ‘no response’ should therefore be questioned. The methodological limitations of using ‘response’ as an outcome has been investigated in a valid study by Kirsch et al. who conclude that: “response rates based on continuous data do not add information, and they can create an illusion of clinical effectiveness” [202]. In retrospect, due to these methodological limitations we should not have assessed ‘no remission’ or ‘no response’ as outcomes. This is a clear limitation of our review [16, 199–201].

Our tests for subgroup difference comparing trials with a baseline HDRS score below and above 23 points and meta-regression showed that the effects of SSRIs seem to increase with increased baseline HDRS score. Others have also shown that trials randomising participants with a higher baseline HDRS mean average seem to show larger effects of antidepressants [7, 8]. However, it is difficult to interpret why trials with higher average baseline HDRS score seem to have a larger effect of SSRIs. This might just be due to random error. No matter, it cannot be concluded based on these results that SSRIs work better on more severely depressed patients. To make such a conclusion individual patient data would be necessary, i.e., it would be necessary to show that it is actually the patients with higher baseline HDRS scores who have the larger effects. Gibbons et al. used longitudinal person-level data from a large set of published and unpublished studies and showed baseline severity was not significantly related to degree of SSRI treatment advantage over placebo [3]. It must be noted that the intervention effects in the group with HDRS scores above 23 points were still below our threshold for clinical significance, supporting Gibbons and co-workers’ results.

Leucht et al. have suggested that effects sizes of SSRIs in randomised clinical trials have declined over time [203]. Post-hoc meta-regression of the HDRS results confirmed their results (effect sizes going down from around 0.8 in the early 1980s to 0.25 in 2012). The reasons for the decreasing effect is not entirely understood but might be due to better methodology nowadays or recruitment of different types of participants [203]. Leucht et al. also suggested that a lack of difference between antidepressants and placebo is caused by an increasing ‘placebo’ effect (spontaneous recovery) [203]. This seem less important from a patient perspective, i.e., whether a certain drug should be used should be based on the benefits and harms of this drug compared with placebo. Furthermore, the increasing placebo effect has recently been severely questioned [188].

Our present systematic review has several strengths. Our protocol was registered prior to the systematic literature search in all relevant databases, data extraction, and data analyses [14]. Data were double-extracted by independent authors minimising the risk of inaccurate data extraction, and we assessed the risk of bias in all trials according to Cochrane [16]. We used Trial Sequential Analysis to control the risks of random errors [25, 29, 204], and the analyses of the primary outcomes showed that the accrued information sizes were sufficient. Both visual assessments of forest plots and statistical test showed limited signs of statistical heterogeneity, e.g., I^2^ was 0% when assessing risk of serious adverse events. Hence, these findings increase the validity of our review results and indicate that the effects shown are consistent across the different trials. Multiple previous reviews and meta-analyses have, as mentioned in our Background, assessed the effects of SSRIs and have generally concluded that SSRIs have significant effects on depressive symptoms [3–8]. However, the estimated results (and not the conclusions the review authors made) of these reviews and meta-analyses actually are in agreement with our present results and show that SSRIs do not seem to benefit patients more than a few HDRS points. This increases the validity of our present results. Furthermore, we assessed in detail the risks of serious adverse events and of non-serious adverse events and found that both were significantly increased by SSRIs.

Our systematic review has several limitations. Our HDRS mean differences were averaged effects. Hence, it cannot be concluded that SSRIs do not have clinically significant effects on all depressed participant. E.g., certain severely depressed patients compared with lightly depressed patients (e.g., so-called professional patients or symptomatic volunteers [203]) might benefit from SSRIs even though there is no evidence backing this hypothesis. However, any clinical research result will have this 'limitation'. Specific patients might benefit from any given intervention even though valid research results have shown that this intervention 'on average' is ineffective or even harmful. All trials were at high risk of bias per several bias risk domains and especially the risk of incomplete outcome data, selective outcome reporting, and insufficient blinding bias may bias our review results. Our GRADE assessments show that due to the high risks of bias the quality of the evidence must be regarded as very low. The high risks of bias question the validity of our meta-analysis results as high risk of bias trials tend to overestimate benefits and underestimate harms [194, 205]. The ‘true’ effect of SSRIs might not even be statistically significant.

We chose to include all SSRIs in our primary analysis. We did this to increase the statistical power and precision and to be able to compare the effects of the different SSRIs in subgroup analysis. Comparing the different SSRIs in test for subgroup differences did not show significant differences, indicating the effects (or lack of effects) of the different SSRIs are similar. Nevertheless, we cannot rule out that certain SSRIs may have beneficial or harmful effects that we have not identified in this review due to lack of relevant data. We identified very limited data on the effects of SSRIs on long-term outcomes, suicidal behaviour, and quality of life, so the effects of SSRIs on these outcomes are unclear. E.g., we only identified six trials assessing quality of life which substantially increase the risk of selective outcome reporting bias and thereby limit the validity of the meta-analysis result. Furthermore, the trialists did not use the same questionnaire. Quality of life is without question an outcome with great relevance to the patient and we urge future trialists to assess quality of life. However, any given quality of life questionnaire must be validated (shown to be correlated to, e.g., suicidal behaviour or other clinical events) before valid conclusions may be drawn based on this outcome. It must be shown that scores on a given questionnaire do reflect the actual ‘quality of life’. Valid consensus on choosing the optimal quality of life assessment method does not exist and this is a limitation of assessing quality of life in depressed patients. Our eight-step procedure used to assess if the thresholds for statistical and clinical significance are crossed, is based on generally accepted and validated methodology but the use of the eight-step procedure has not yet been validated in simulation studies or empirical studies [12, 13]. Even though the eight-step procedure has been used in several systematic reviews it is not universally accepted. This may be a limitation of our methodology.

The Committee for Medicinal Products for Human Use (CHMP) concluded”……… that, as no public health concerns have been identified, no regulatory action is necessary on the basis of Kirsch et al.'s findings” when the latter team questioned the benefits of antidepressants [182]. Per our results, we now believe that there is valid evidence for a public concern regarding the effects of SSRIs. We agree with Andrews et al. that that antidepressants seem to do more harm than good [206]. We have clearly shown that SSRIs significantly increase the risks of both serious and several non-serious adverse events. The observed harmful effects seem to outweigh the potential small beneficial clinical effects of SSRIs, if they exist. Our results confirm the findings from other studies questioning the effects of SSRIs [8, 207], but are in contrast to the results of other reviews concluding that SSRIs are effective interventions for depression [3, 6, 10, 208]. However, our present analyses represent the most comprehensive systematic review on the topic and we hope it may guide clinical practice.

## Conclusions

SSRIs versus placebo seem to have statistically significant effects on depressive symptoms, but the clinical significance of these effects seems questionable and all trials were at high risk of bias. Furthermore, SSRIs versus placebo significantly increase the risk of both serious and non-serious adverse events. Our results show that the harmful effects of SSRIs versus placebo for major depressive disorder seem to outweigh any potentially small beneficial effects.

## Additional files

Additional file 1: Search strategies. (DOC 41 kb)

Additional file 2: List of excluded trials. (TXT 2726 kb)

Additional file 3: Table S1. Trial characteristics. (XLS 80 kb)

Additional file 4: Figure S1. Subgroup analysis of for profit bias. (PNG 56 kb)

Additional file 5: Summary of findings table. (DOCX 72 kb)

Additional file 6: Summary of the 20 Most Common Adverse Events in the Included Trials. (PDF 37 kb)

Additional file 7: Figure S3. Meta-analysis of abnormal ejaculation. (PDF 203 kb)

Additional file 8: Figure S4. Meta-analysis of tremor. (PDF 240 kb)

Additional file 9: Figure S5. Meta-analysis of anorexia. (PDF 194 kb)

Additional file 10: Figure S6. Meta-analysis of nausea. (PDF 579 kb)

Additional file 11: Figure S7. Meta-analysis of somnolence. (PDF 459 kb)

Additional file 12: Figure S8. Meta-analysis of sweating. (PDF 283 kb)

Additional file 13: Figure S9. Meta-analysis of asthenia. (PDF 234 kb)

Additional file 14: Figure S10. Meta-analysis of diarrhoea. (PDF 475 kb)

Additional file 15: Figure S11. Meta-analysis of constipation. (PDF 369 kb)

Additional file 16: Figure S12. Meta-analysis of insomnia. (PDF 538 kb)

Additional file 17: Figure S13. Meta-analysis of dizziness. (PDF 406 kb)

Additional file 18 Figure S14. Meta-analysis of dry mouth. (PDF 542 kb)

Additional file 19: Figure S15. Meta-analysis of libido decreased. (PDF 136 kb)

Additional file 20: Figure S16. Meta-analysis of sexual dysfunction. (PDF 124 kb)

Additional file 21: Figure S17. Meta-analysis of appetite decreased. (PDF 123 kb)

Additional file 22: Figure S18. Meta-analysis of fatigue. (PDF 239 kb)

Additional file 23: Figure S19. Meta-analysis of vomiting or stomach upset. (PDF 190 kb)

Additional file 24: Figure S20. Meta-analysis of flu syndrome. (PDF 130 kb)

Additional file 25: Figure S21. Meta-analysis of drowsiness. (PDF 104 kb)

Additional file 26: Figure S22. Meta-analysis of blurred or abnormal vision. (PDF 166 kb)

Additional file 27: Figure S23. Meta-analysis of nervousness. (PDF 224 kb)

Additional file 28: Figure S24. Meta-analysis of back pain. (PDF 153 kb)

Additional file 29: Figure S25. Meta-analysis of headache.

Additional file 30: Figure S26 Meta-analysis of dyspepsia. (PDF 226 kb)

Additional file 31: Figure S27 Meta-analysis of weight loss. (PDF 97 kb)

Additional file 32: Figure S2 Meta-analysis of no response. (PNG 105 kb)
