# Erratum to: Selective serotonin reuptake inhibitors versus placebo in patients with major depressive disorder. A systematic review with meta-analysis and Trial Sequential Analysis

**DOI:** 10.1186/s12888-017-1311-5

**Published:** 2017-05-03

**Authors:** Janus Christian Jakobsen, Kiran Kumar Katakam, Anne Schou, Signe Gade Hellmuth, Sandra Elkjær Stallknecht, Katja Leth-Møller, Maria Iversen, Marianne Bjørnø Banke, Iggiannguaq Juhl Petersen, Sarah Louise Klingenberg, Jesper Krogh, Sebastian Elgaard Ebert, Anne Timm, Jane Lindschou, Christian Gluud

**Affiliations:** 1 The Copenhagen Trial Unit, Centre for Clinical Intervention Research, Department 7812 Rigshospitalet, Copenhagen University Hospital, Blegdamsvej 9, Rigshospitalet, 2100 Copenhagen, DK Denmark; 2 Department of Cardiology, Holbæk Hospital, Holbæk, Denmark; 3 Mental Health Centre Copenhagen, Faculty of Health Sciences, University of Copenhagen, Copenhagen, Denmark

## Erratum

After the publication of this paper the authors noticed a few errors in the text. There are outlines below:

1. Results section, under Hamilton depression rating scale (HDRS): In the sentence ‘**Nineteen** trials reported only mean HDRS change scores or presented a graph showing the mean change HDRS scores, but did not report the SD’ **Eighteen** should replace **Nineteen**
2. Results section, under Hamilton depression rating scale (HDRS):In the sentence ‘Random-effects meta-analysis of the results of all **92** trials showed that SSRIs versus placebo significantly reduced the HDRS score (mean difference −2.25 points; 95% CI −2.69 to −1.83; P<0.00001)’ **91** should replace **92**
3. Results section, under Other subgroup analyses: In the sentence ‘Meta-analysis of the results of the **26** trials with a mean baseline HDRS score >23 points showed a mean difference of −2.69 HDRS points; 95% CI −3.59 to −1.78; P<0.00001.’ **25** should replace **26**
4. Table 2 [Summary of serious adverse events in the included trials] ‘**Nemroff** et al., **2005**’ should read ‘**Nemeroff** et al., **2007’** and in the same line ‘**Fluxetine**’ should read ‘**Fluoxetine**’
