# Supplementary material for: Selective serotonin reuptake inhibitors versus placebo in patients with major depressive disorder. A systematic review with meta-analysis and Trial Sequential Analysis
Source: BMC Psychiatry. 2017 Feb 8;17:58. doi: 10.1186/s12888-016-1173-2 (PMC5299662; doi:10.1186/s12888-016-1173-2)
Supplement: Supplementary file 1 — Search strategies. (DOC 41 kb) [file 12888_2016_1173_MOESM1_ESM.doc]

**Search strategies for**

**Antidepressiva review**

**(J Jakobsen)**

**Searches performed 28 May 2015.**

**Total number of references identified: 24853 references**

**Number of duplicates excluded: 9569 references**

**Number of references in final list: 15284 references**

**BATCH NAME:** 150528_J Jakobsen_Antidepressiva Total Hits

# Cochrane Central Register of Controlled Trials (CENTRAL) (Issue 4 of 12, 2015) (2200 hits in CENTRAL)

#1 MeSH descriptor: [Depressive Disorder] explode all trees

#2 MeSH descriptor: [Depression] explode all trees

#3 MeSH descriptor: [Affective Symptoms] explode all trees

#4 #1 or #2 or #3

#5 MeSH descriptor: [Citalopram] explode all trees

#6 MeSH descriptor: [Sertraline] explode all trees

#7 MeSH descriptor: [Fluoxetine] explode all trees

#8 MeSH descriptor: [Paroxetine] explode all trees

#9 MeSH descriptor: [Fluvoxamine] explode all trees

#10 citalopram or celexa or cipra* or Escitalopram or lexapro or seroplex or lexam* or esertia or sertraline or zoloft or lustral or fluoxetine or prozac or rapiflux or serafem or selfemra or fontex or paroxetine or paxil or pexeva or aropax or seroxat or sereupin or fluvoxamine or luvox or fevarin or dumirox or venlafaxine or ef*exor or duloxetine or cymbalta or ariclaim or xeristar or yentreve or duzela or mirtazapine or remeron or avanza or zispin or miro or combar

#11 #5 or #6 or #7 or #8 or #9 or #10

#12 #4 and #11

**MEDLINE (Ovid SP) (1946 to May 2015) (3614 hits)**

1. exp Depressive Disorder/

2. exp Depression/

3. exp Affective Symptoms/

4. 1 or 2 or 3

5. exp Citalopram/

6. exp Sertraline/

7. exp Fluoxetine/

8. exp Paroxetine/

9. exp Fluvoxamine/

10. (citalopram or celexa or cipra* or Escitalopram or lexapro or seroplex or lexam* or esertia or sertraline or zoloft or lustral or fluoxetine or prozac or rapiflux or serafem or selfemra or fontex or paroxetine or paxil or pexeva or aropax or seroxat or sereupin or fluvoxamine or luvox or fevarin or dumirox or venlafaxine or ef*exor or duloxetine or cymbalta or ariclaim or xeristar or yentreve or duzela or mirtazapine or remeron or avanza or zispin or miro or combar).mp. [mp=title, abstract, original title, name of substance word, subject heading word, keyword heading word, protocol supplementary concept word, rare disease supplementary concept word, unique identifier]

11. 5 or 6 or 7 or 8 or 9 or 10

12. 4 and 11

13. (random* or blind* or placebo* or meta-analys*).mp. [mp=title, abstract, original title, name of substance word, subject heading word, keyword heading word, protocol supplementary concept word, rare disease supplementary concept word, unique identifier]

15. 12 and 13

**EMBASE (Ovid SP) (1974 to May 2015) (11291hits)**

1. exp depression/

2. exp emotional disorder/

3. 1 or 2

4. exp citalopram/

5. exp sertraline/

6. exp fluoxetine/

7. exp paroxetine/

8. exp fluvoxamine/

9. exp venlafaxine/

10. exp duloxetine/

11. exp mirtazapine/

12. (citalopram or celexa or cipra* or Escitalopram or lexapro or seroplex or lexam* or esertia or sertraline or zoloft or lustral or fluoxetine or prozac or rapiflux or serafem or selfemra or fontex or paroxetine or paxil or pexeva or aropax or seroxat or sereupin or fluvoxamine or luvox or fevarin or dumirox or venlafaxine or ef*exor or duloxetine or cymbalta or ariclaim or xeristar or yentreve or duzela or mirtazapine or remeron or avanza or zispin or miro or combar).mp. [mp=title, abstract, subject headings, heading word, drug trade name, original title, device manufacturer, drug manufacturer, device trade name, keyword]

13. 4 or 5 or 6 or 7 or 8 or 9 or 10 or 11 or 12

14. 3 and 13

15. (random* or blind* or placebo* or meta-analys*).mp. [mp=title, abstract, subject headings, heading word, drug trade name, original title, device manufacturer, drug manufacturer, device trade name, keyword]

17. 14 and 15

**Science Citation Index Expanded (1900 to May 2015) (5131 hits)**#5 5,131 #4 AND #3

#4 1,344,031 TS=(random* or blind* or placebo* or meta-analys*)

#3 10,243 #2 AND #1

#2 34,048 TS=(citalopram or celexa or cipra* or Escitalopram or lexapro or seroplex or lexam* or esertia or sertraline or zoloft or lustral or fluoxetine or prozac or rapiflux or serafem or selfemra or fontex or paroxetine or paxil or pexeva or aropax or seroxat or sereupin or fluvoxamine or luvox or fevarin or dumirox or venlafaxine or ef*exor or duloxetine or cymbalta or ariclaim or xeristar or yentreve or duzela or mirtazapine or remeron or avanza or zispin or miro or combar)

#1 148,257 TS=((depressi* OR affective* OR dysthymic) AND (disorder* OR symptom*))

**PsycINFO (1806 to May 2015) (2617 hits)**

1. exp Affective Disorders/

2. exp "Depression (Emotion)"/

3. exp atypical depression/

4. 1 or 2 or 3

5. exp Citalopram/

6. exp Sertraline/

7. exp Fluoxetine/

8. exp Paroxetine/

9. exp Fluvoxamine/

10. exp Venlafaxine/

11. (citalopram or celexa or cipra* or Escitalopram or lexapro or seroplex or lexam* or esertia or sertraline or zoloft or lustral or fluoxetine or prozac or rapiflux or serafem or selfemra or fontex or paroxetine or paxil or pexeva or aropax or seroxat or sereupin or fluvoxamine or luvox or fevarin or dumirox or venlafaxine or ef*exor or duloxetine or cymbalta or ariclaim or xeristar or yentreve or duzela or mirtazapine or remeron or avanza or zispin or miro or combar).mp. [mp=title, abstract, heading word, table of contents, key concepts, original title, tests & measures]

12. 5 or 6 or 7 or 8 or 9 or 10 or 11

13. 4 and 12

14. (random* or blind* or placebo* or meta-analys*).mp. [mp=title, abstract, heading word, table of contents, key concepts, original title, tests & measures]

15. 13 and 14
