# Supplementary figures and images for: Selective serotonin reuptake inhibitors versus placebo in patients with major depressive disorder. A systematic review with meta-analysis and Trial Sequential Analysis
Source: BMC Psychiatry. 2017 Feb 8;17:58. doi: 10.1186/s12888-016-1173-2 (PMC5299662; doi:10.1186/s12888-016-1173-2)

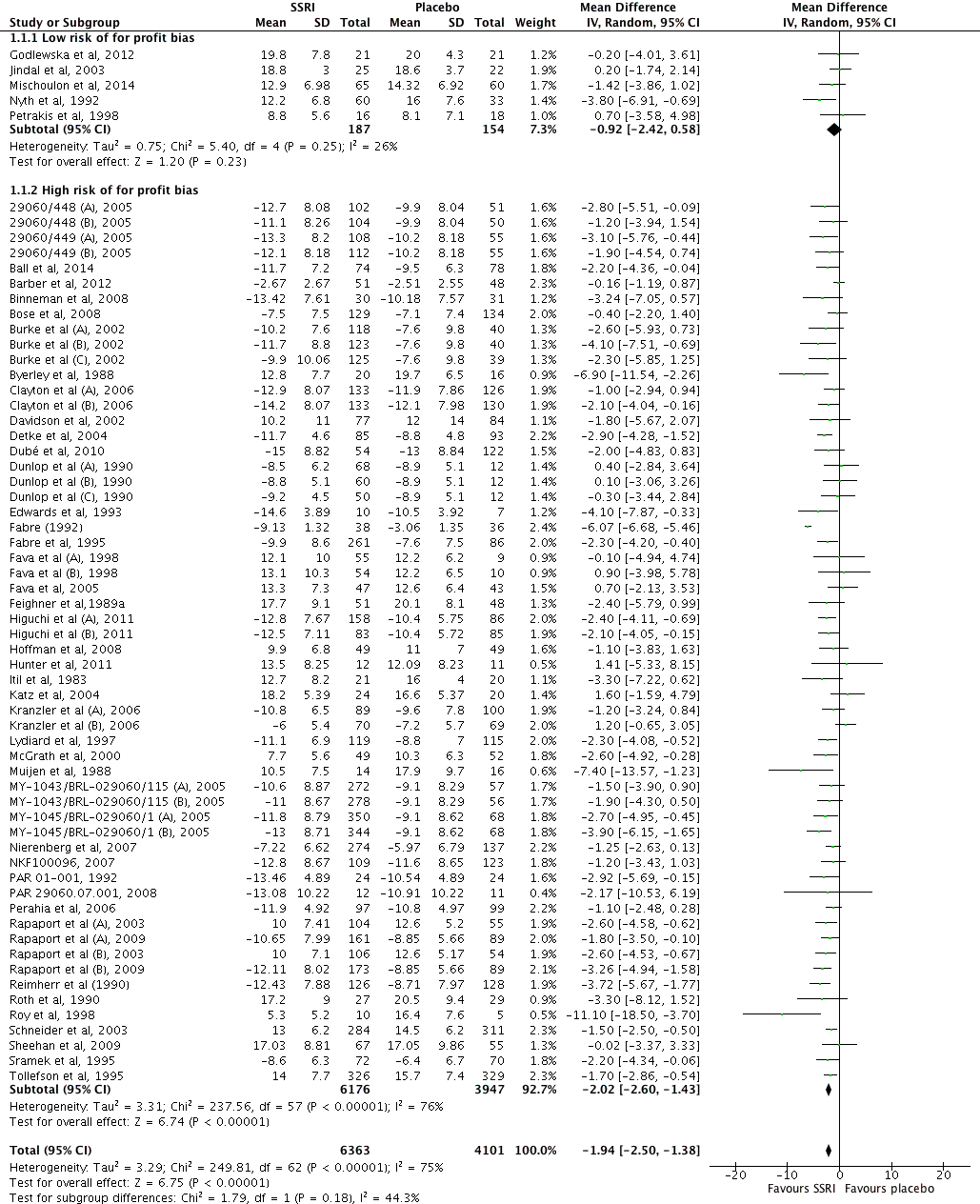

Supplement: Supplementary file 4 — Subgroup analysis of for profit bias. (PNG 56 kb) [file 12888_2016_1173_MOESM4_ESM.png]

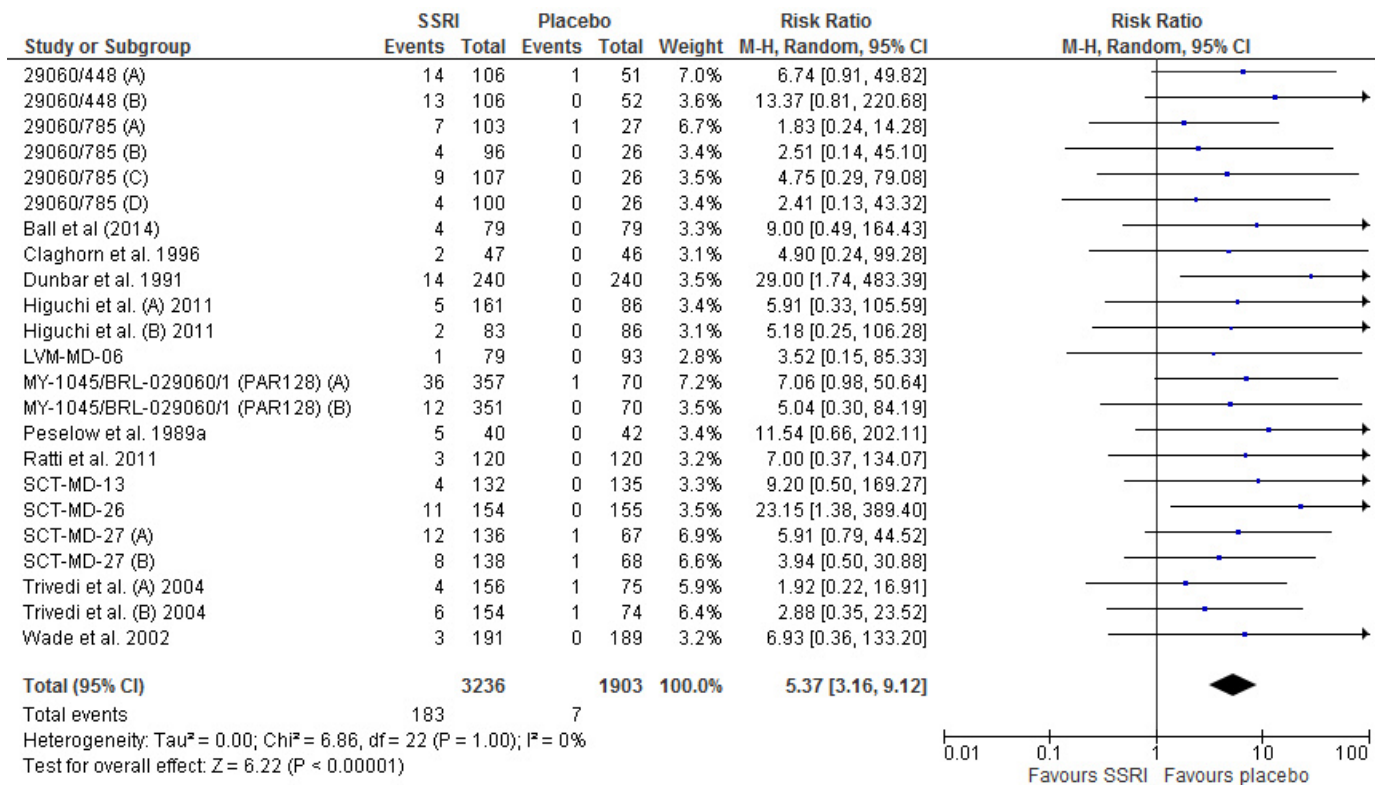

Supplement: Supplementary file 7 — Meta-analysis of abnormal ejaculation. (PDF 203 kb) [file 12888_2016_1173_MOESM7_ESM.pdf]

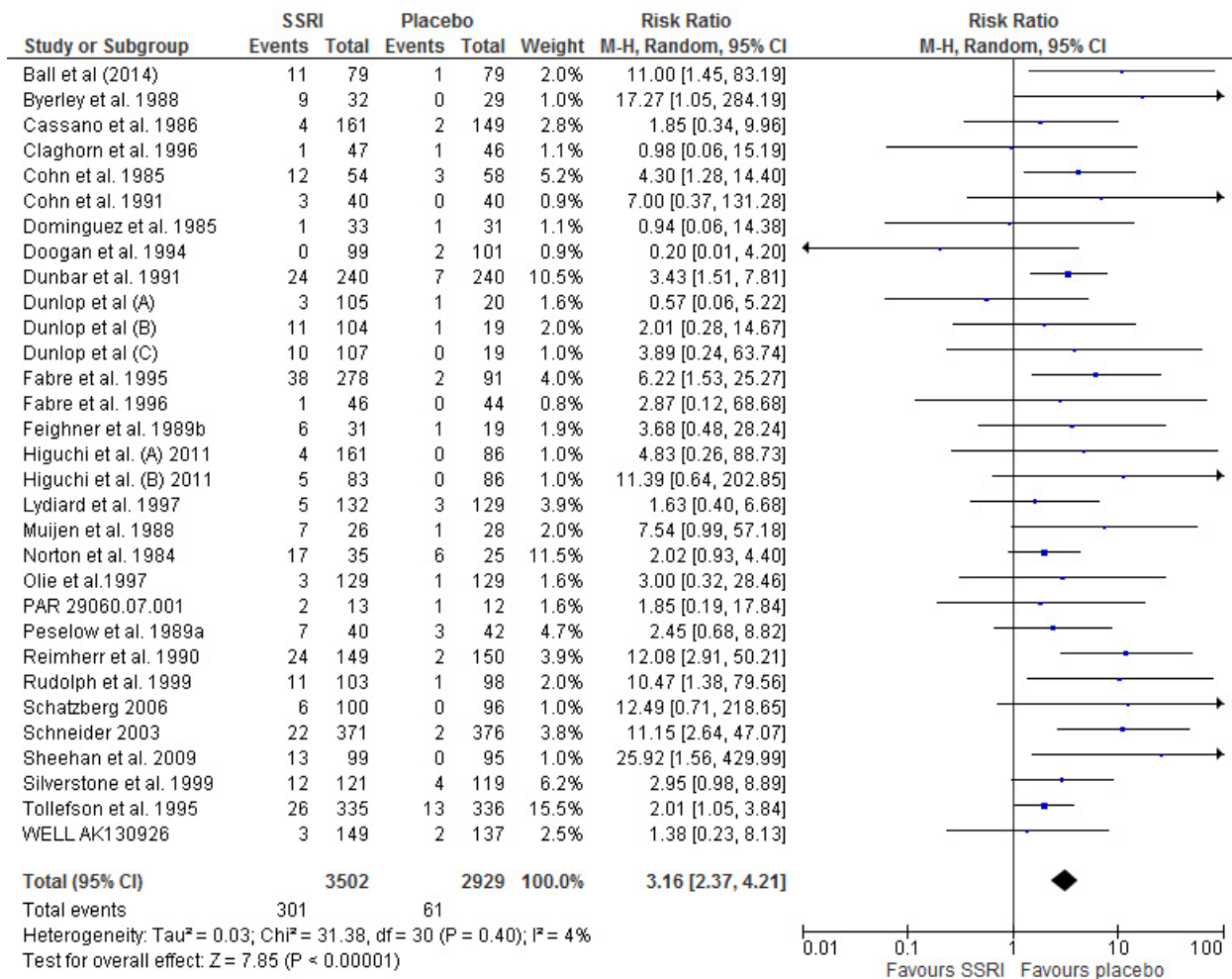

Supplement: Supplementary file 8 — Meta-analysis of tremor. (PDF 240 kb) [file 12888_2016_1173_MOESM8_ESM.pdf]

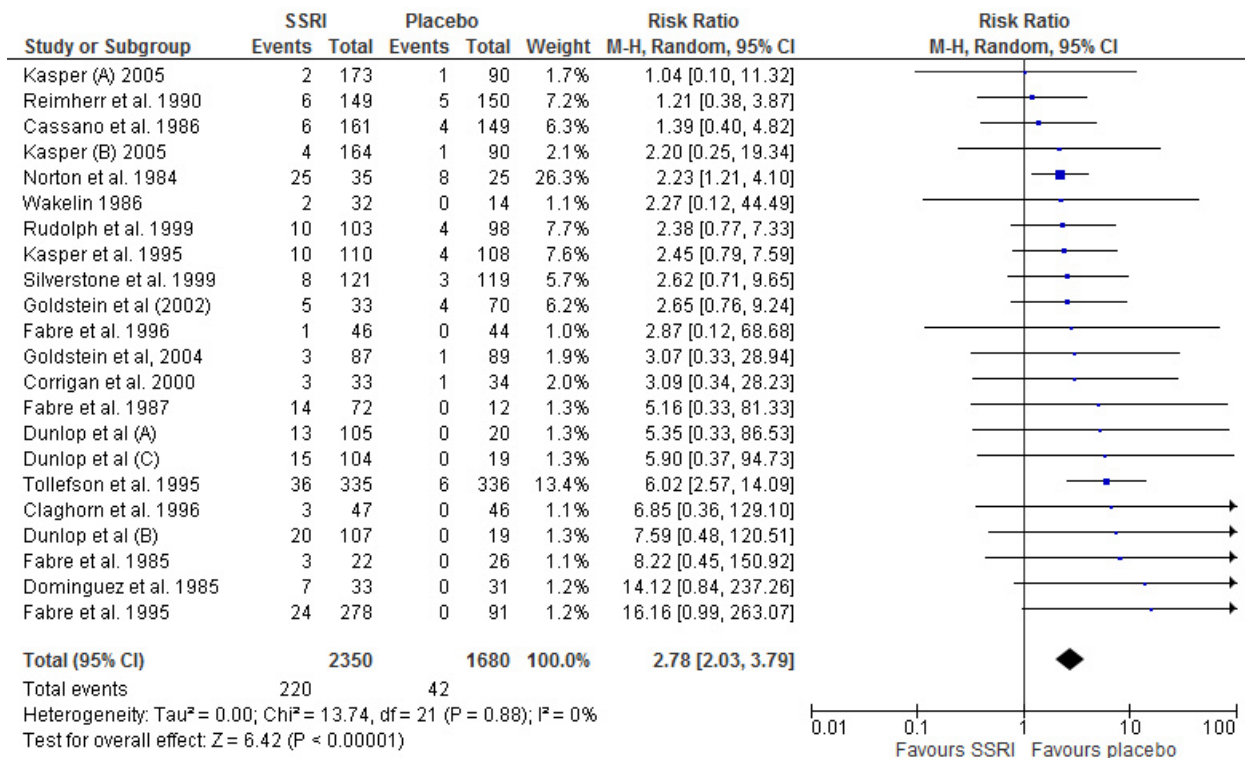

Supplement: Supplementary file 9 — Meta-analysis of anorexia. (PDF 194 kb) [file 12888_2016_1173_MOESM9_ESM.pdf]

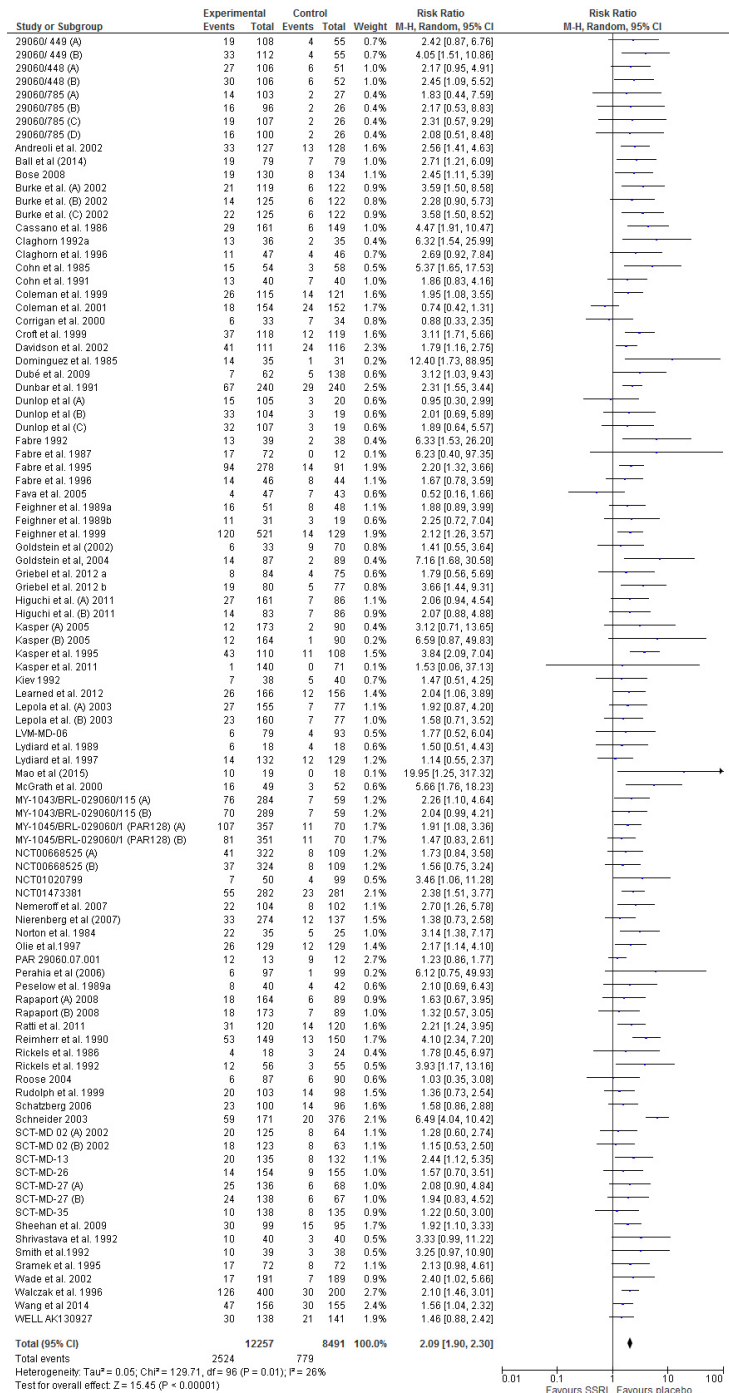

Supplement: Supplementary file 10 — Meta-analysis of nausea. (PDF 579 kb) [file 12888_2016_1173_MOESM10_ESM.pdf]

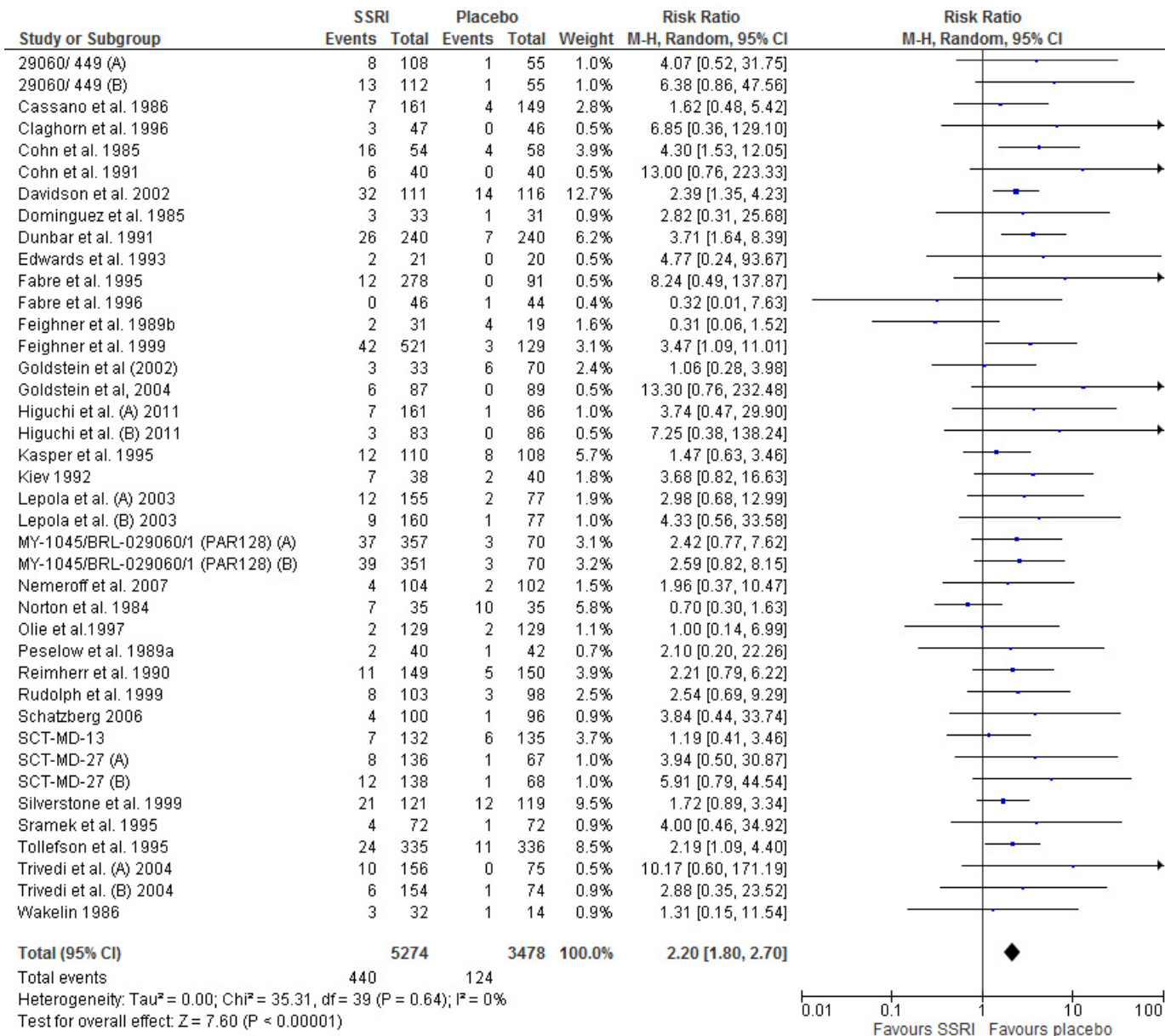

Supplement: Supplementary file 12 — Meta-analysis of sweating. (PDF 283 kb) [file 12888_2016_1173_MOESM12_ESM.pdf]

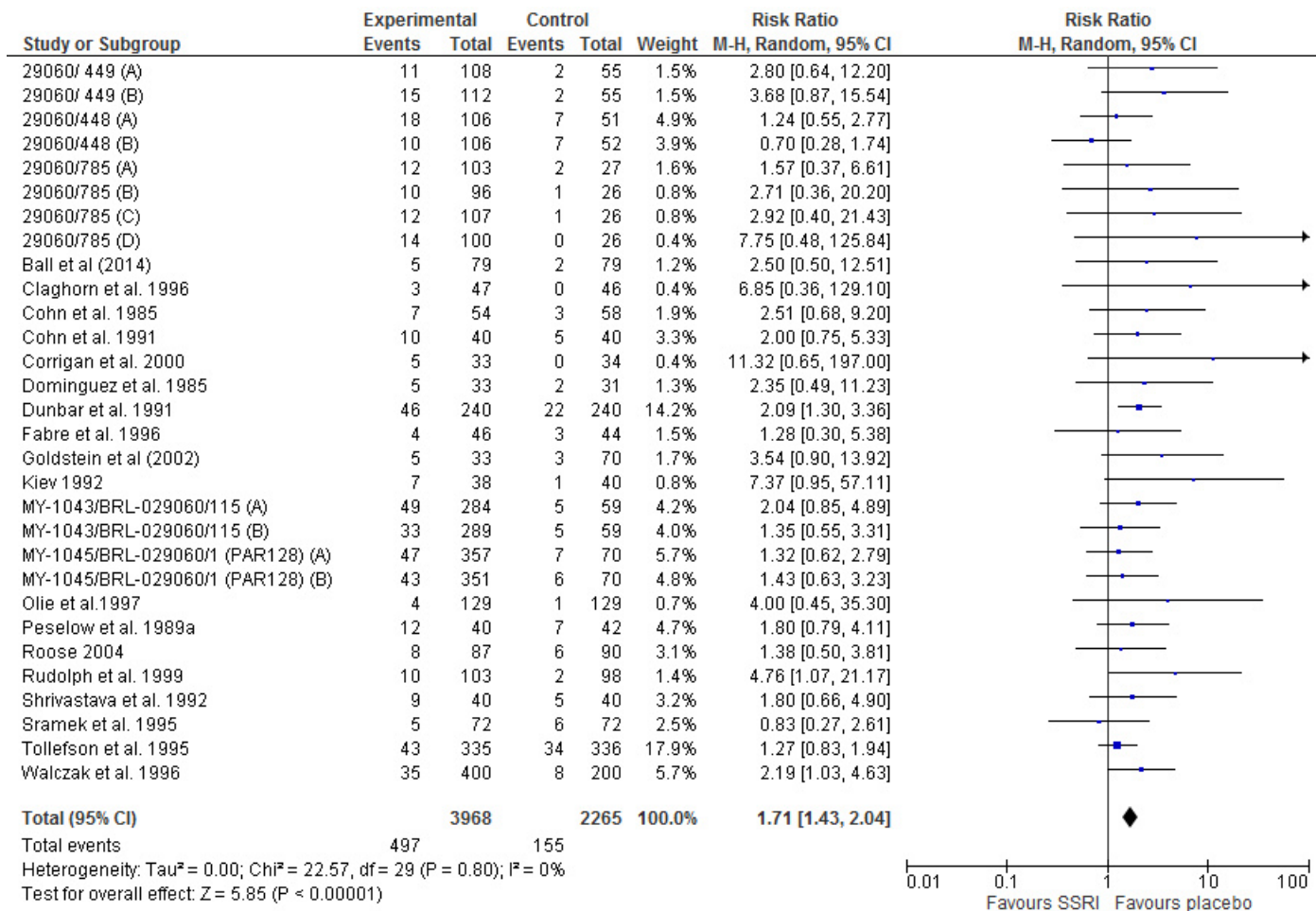

Supplement: Supplementary file 13 — Meta-analysis of asthenia. (PDF 234 kb) [file 12888_2016_1173_MOESM13_ESM.pdf]

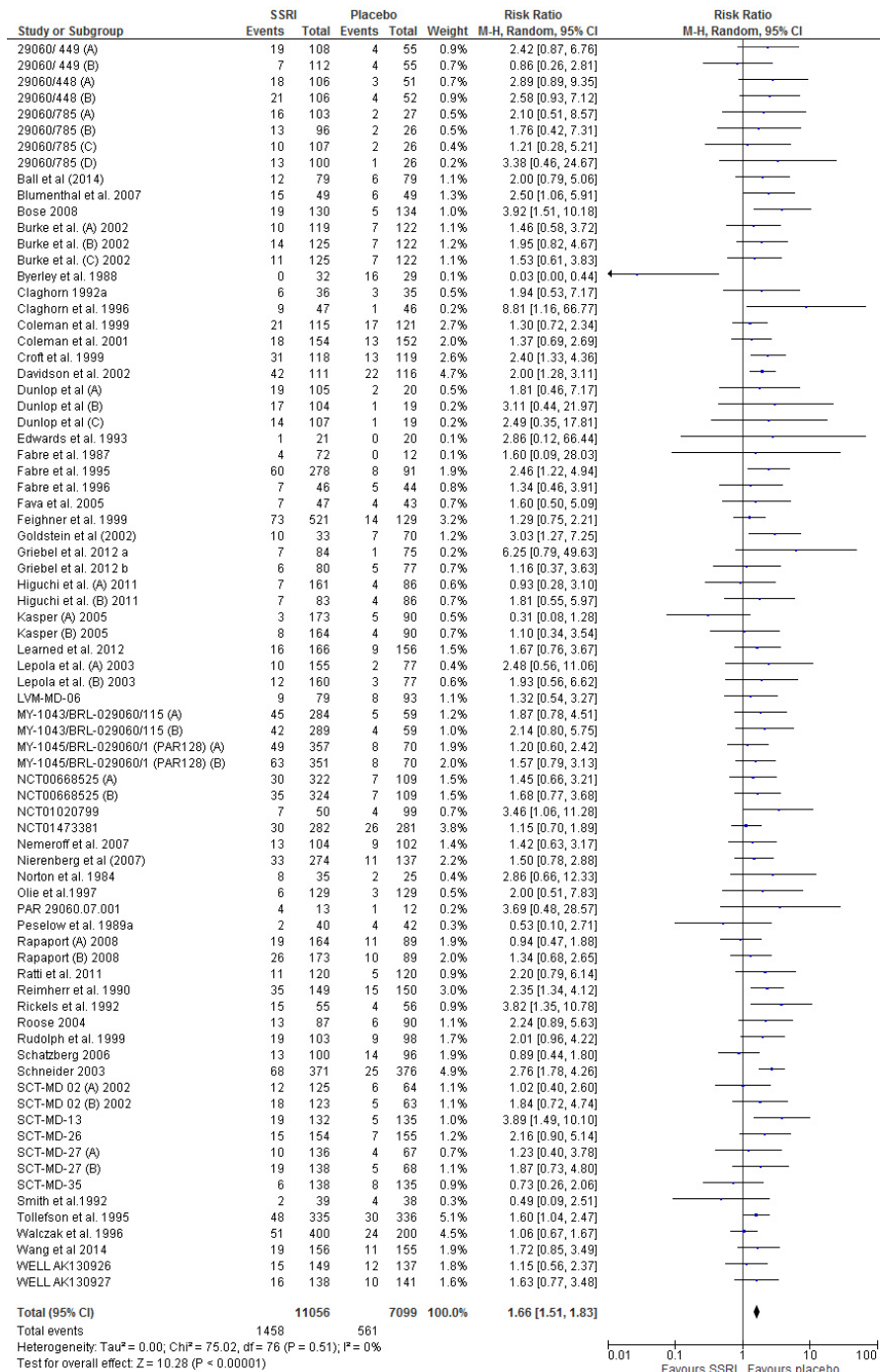

Supplement: Supplementary file 14 — Meta-analysis of diarrhoea. (PDF 475 kb) [file 12888_2016_1173_MOESM14_ESM.pdf]

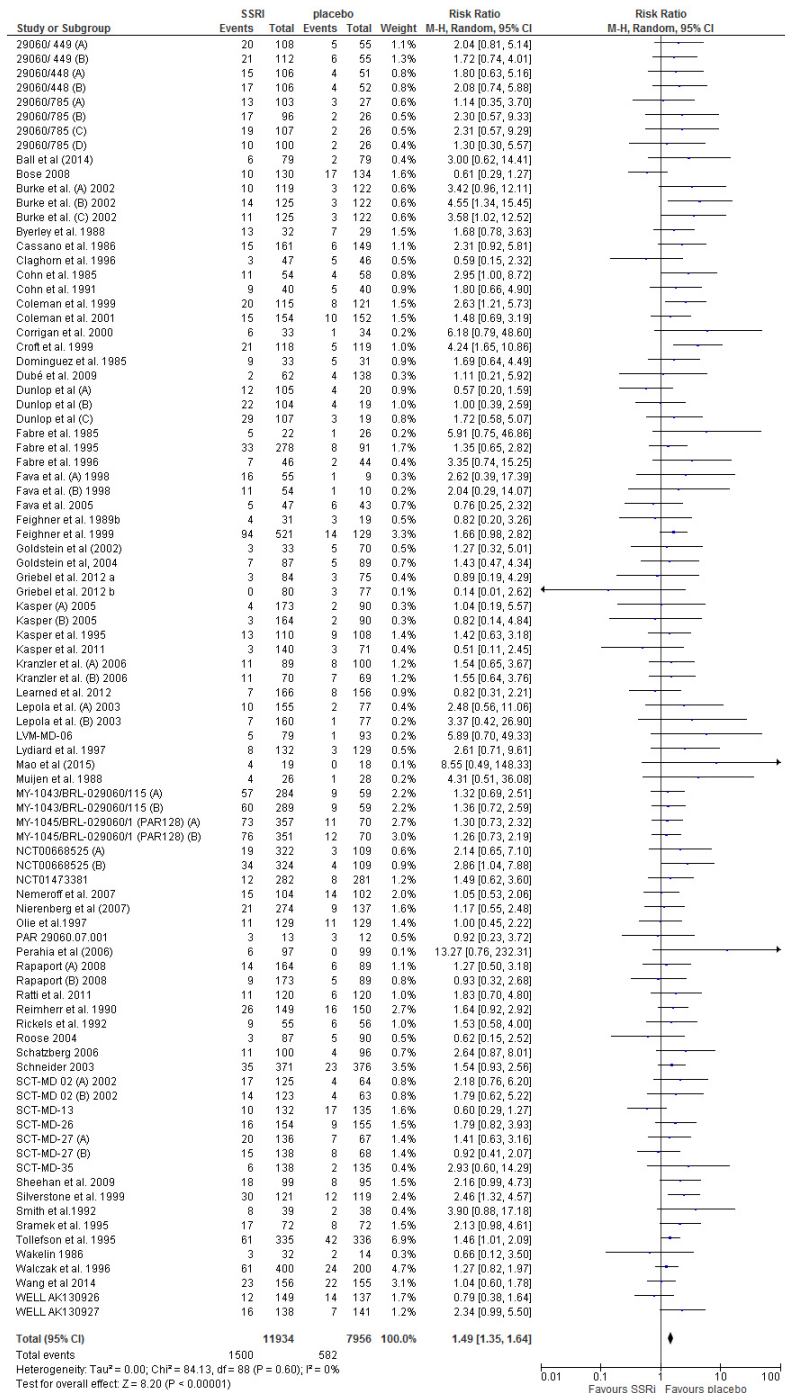

Supplement: Supplementary file 16 — Meta-analysis of insomnia. (PDF 538 kb) [file 12888_2016_1173_MOESM16_ESM.pdf]

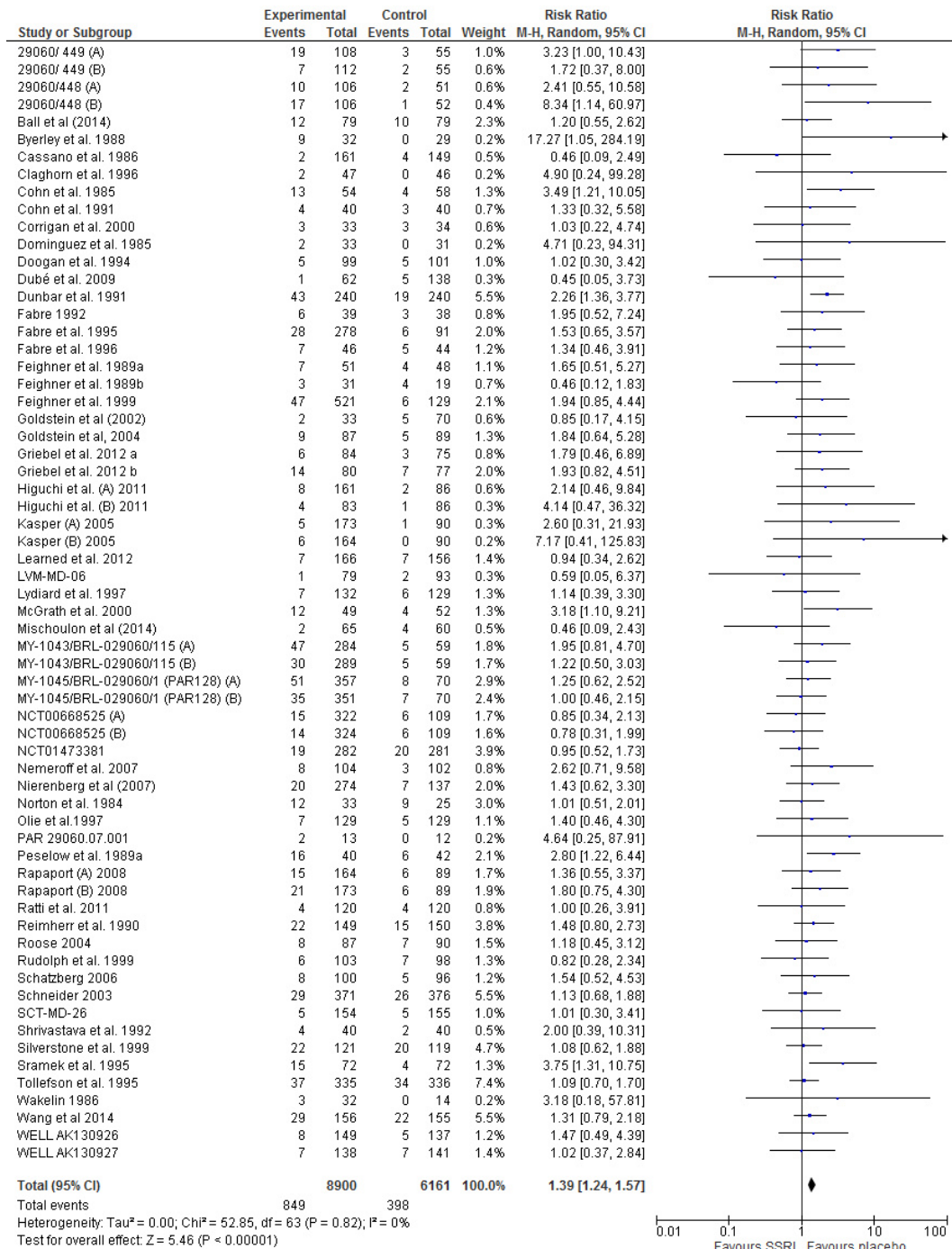

Supplement: Supplementary file 17 — Meta-analysis of dizziness. (PDF 406 kb) [file 12888_2016_1173_MOESM17_ESM.pdf]

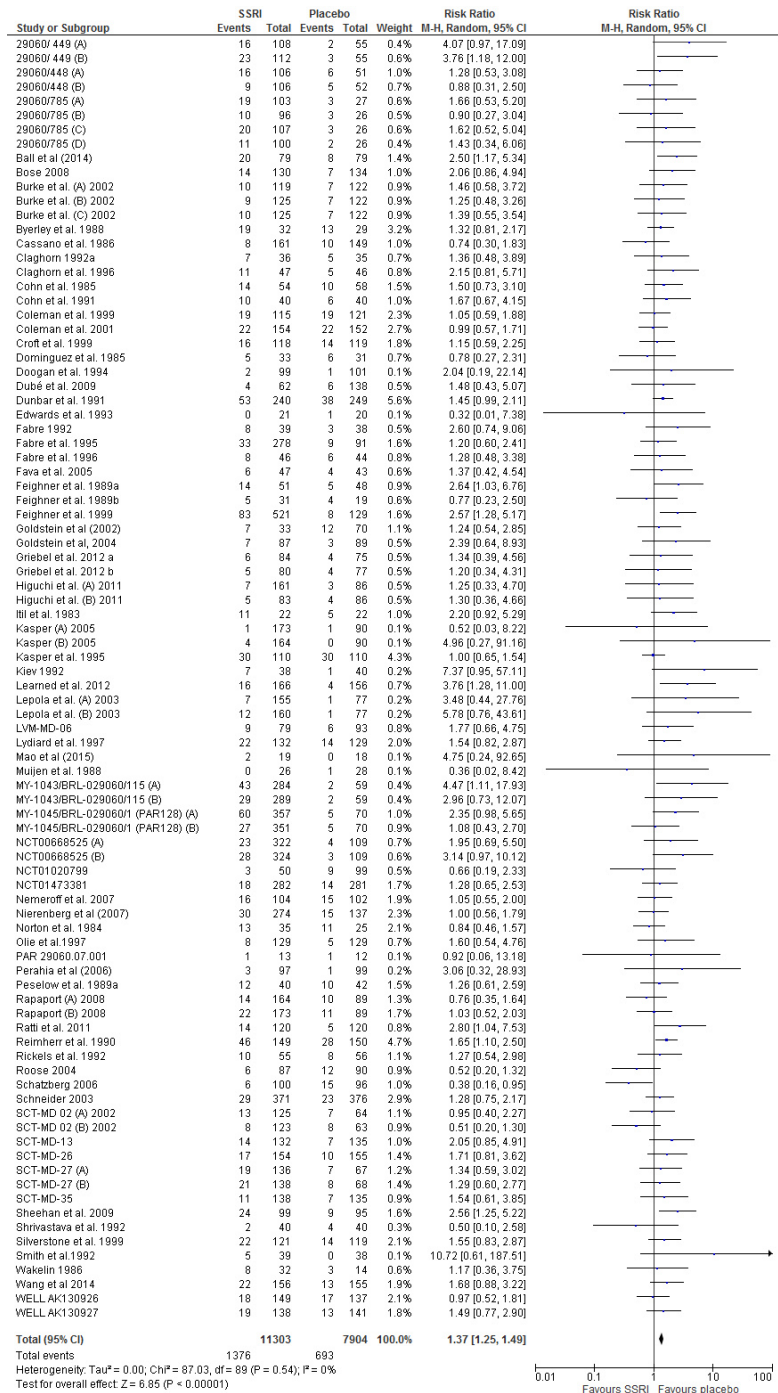

Supplement: Supplementary file 18 — Meta-analysis of dry mouth. (PDF 542 kb) [file 12888_2016_1173_MOESM18_ESM.pdf]

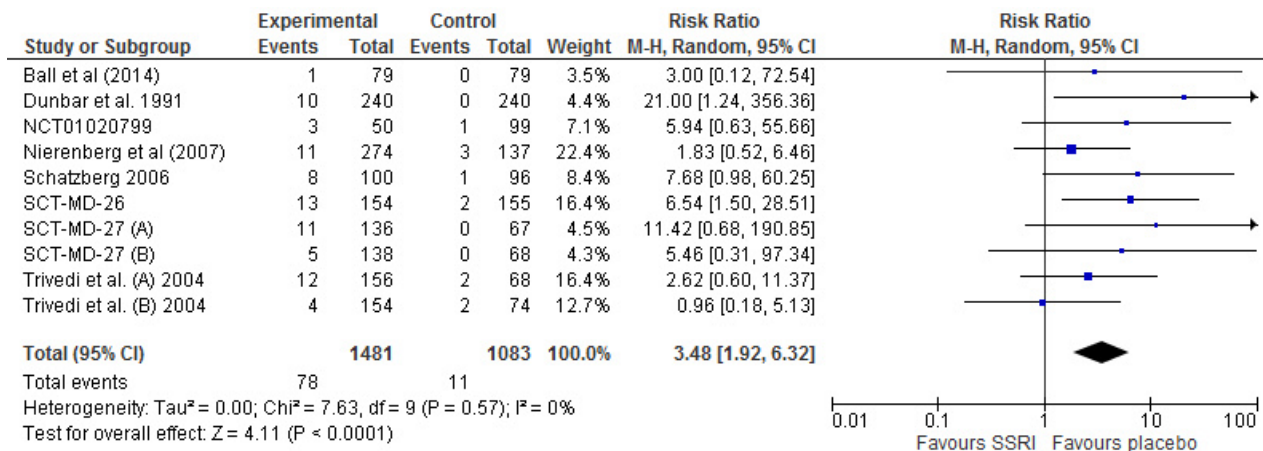

Supplement: Supplementary file 19 — Meta-analysis of libido decreased. (PDF 136 kb) [file 12888_2016_1173_MOESM19_ESM.pdf]

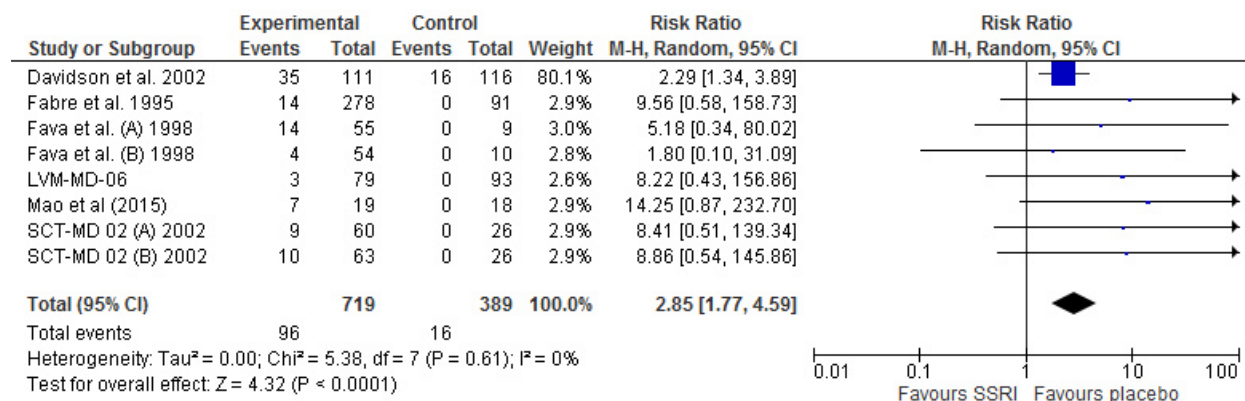

Supplement: Supplementary file 20 — Meta-analysis of sexual dysfunction. (PDF 124 kb) [file 12888_2016_1173_MOESM20_ESM.pdf]

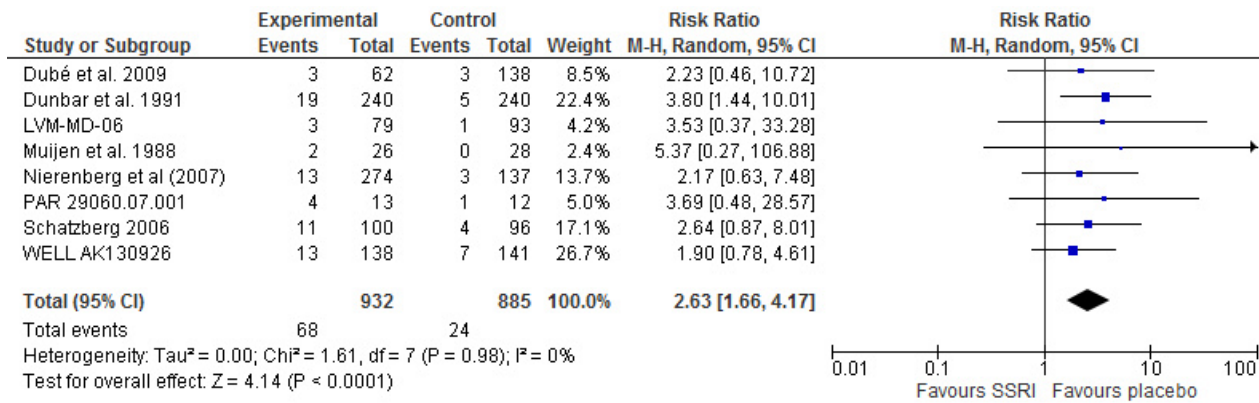

Supplement: Supplementary file 21 — Meta-analysis of appetite decreased. (PDF 123 kb) [file 12888_2016_1173_MOESM21_ESM.pdf]

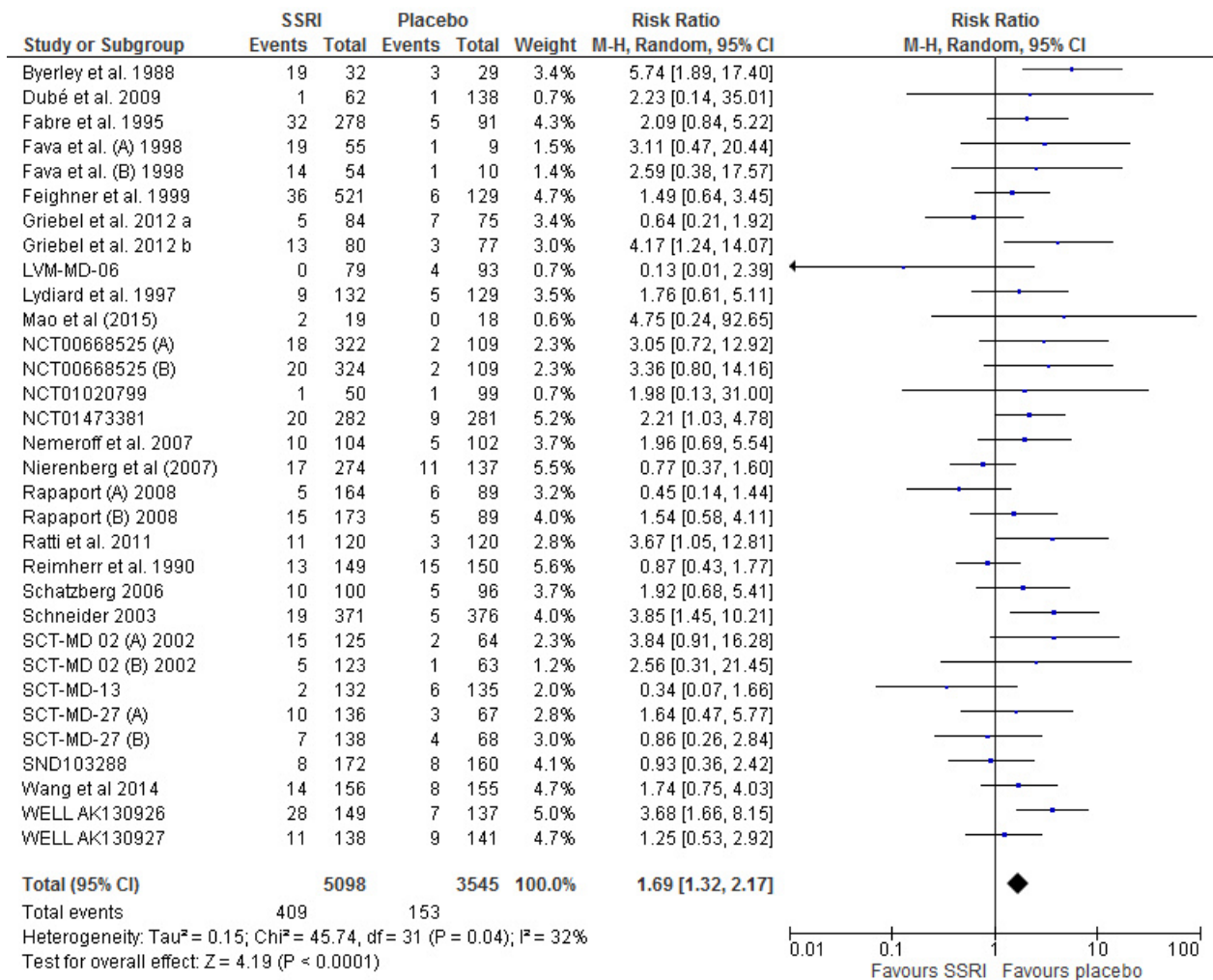

Supplement: Supplementary file 22 — Meta-analysis of fatigue. (PDF 239 kb) [file 12888_2016_1173_MOESM22_ESM.pdf]

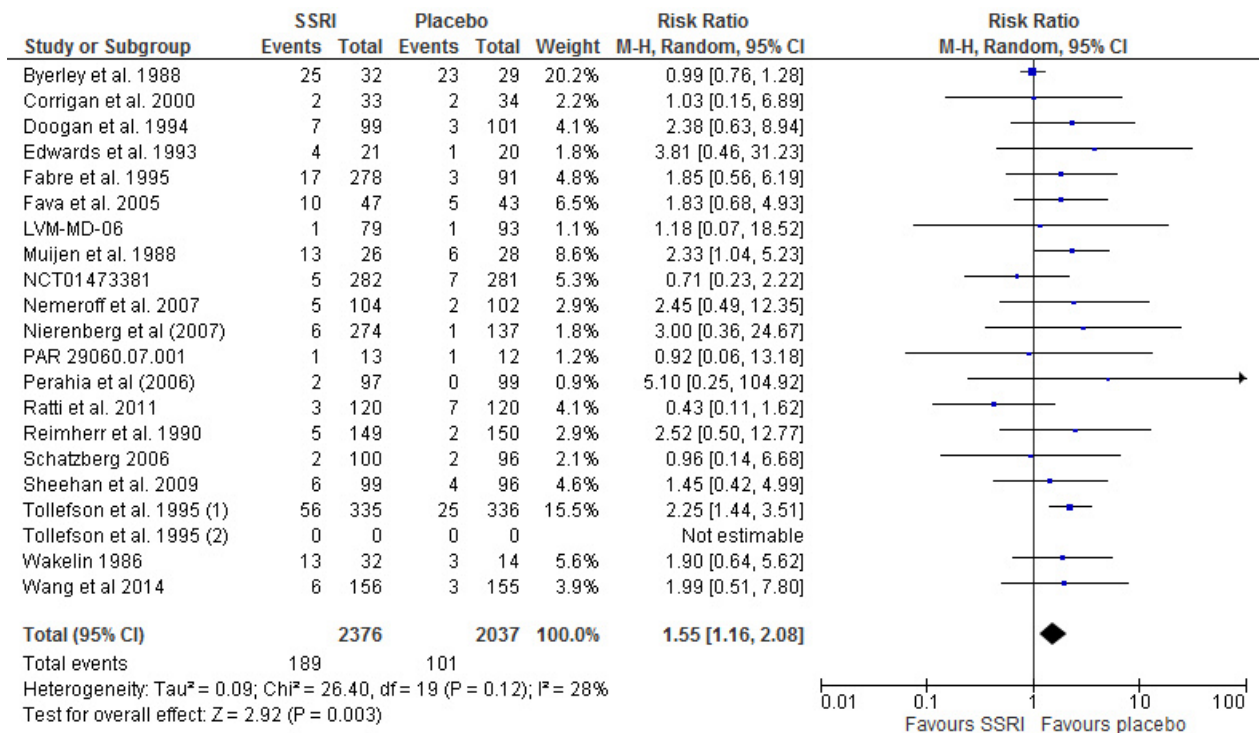

#### Footnotes

(1) nausea

(2) Dyspepsia

Supplement: Supplementary file 23 — Meta-analysis of vomiting or stomach upset. (PDF 190 kb) [file 12888_2016_1173_MOESM23_ESM.pdf]

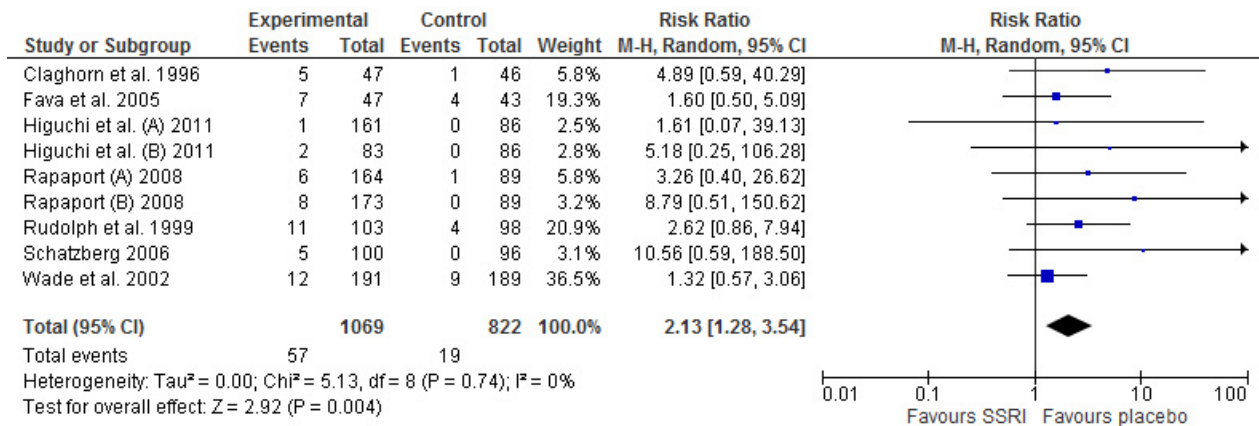

Supplement: Supplementary file 24 — Meta-analysis of flu syndrome. (PDF 130 kb) [file 12888_2016_1173_MOESM24_ESM.pdf]

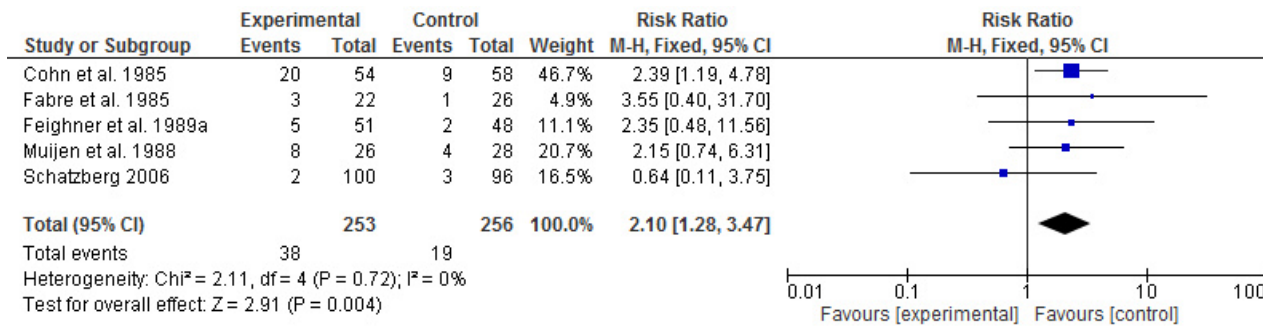

Supplement: Supplementary file 25 — Meta-analysis of drowsiness. (PDF 104 kb) [file 12888_2016_1173_MOESM25_ESM.pdf]

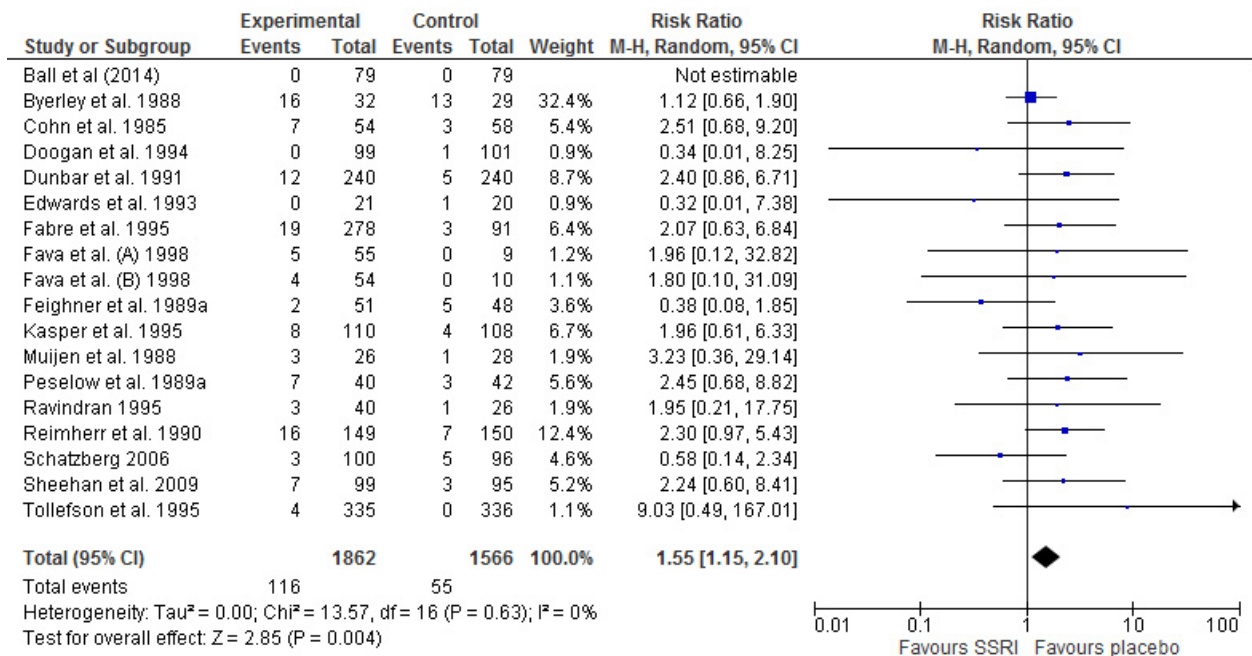

Supplement: Supplementary file 26 — Meta-analysis of blurred or abnormal vision. (PDF 166 kb) [file 12888_2016_1173_MOESM26_ESM.pdf]

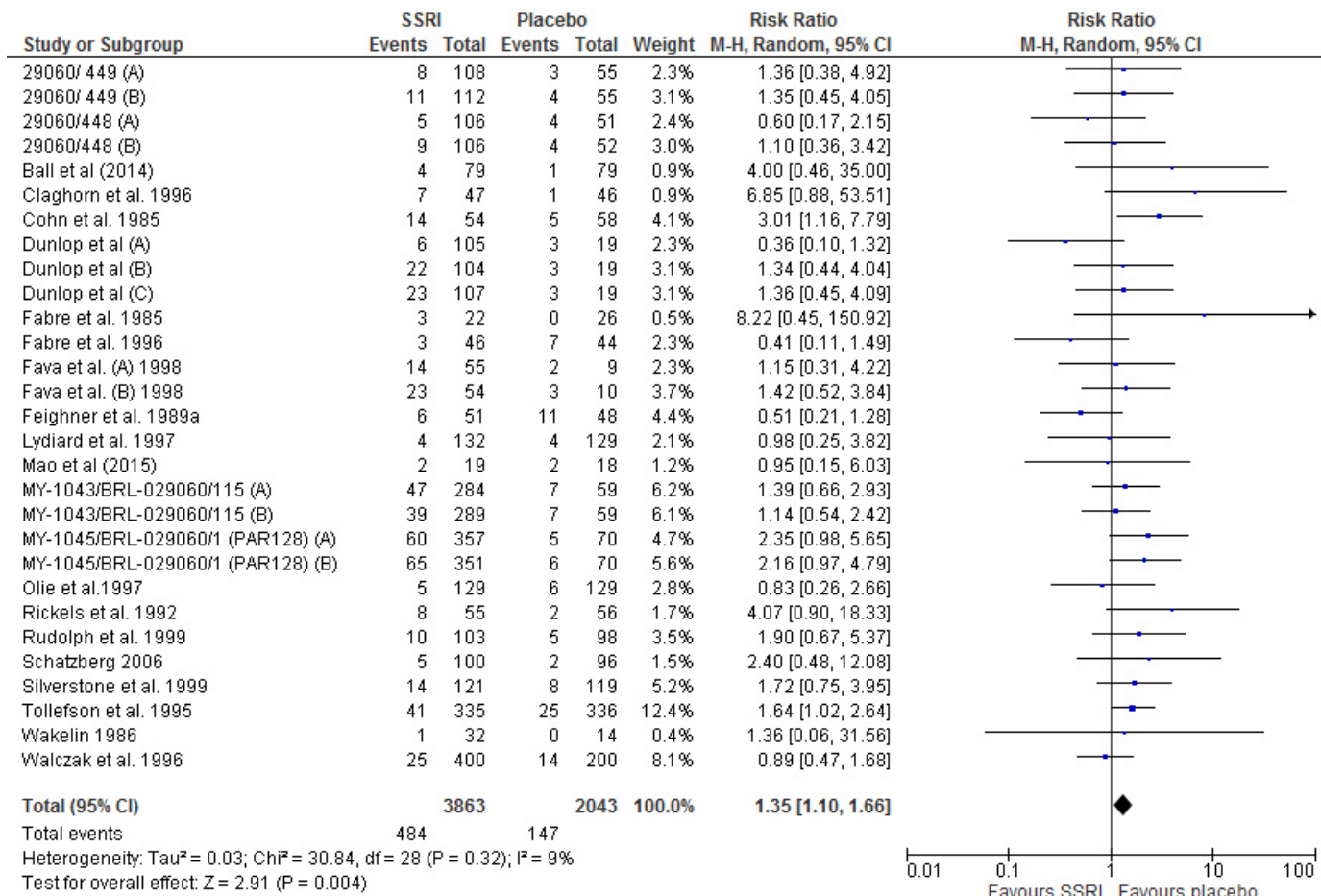

Supplement: Supplementary file 27 — Meta-analysis of nervousness. (PDF 224 kb) [file 12888_2016_1173_MOESM27_ESM.pdf]

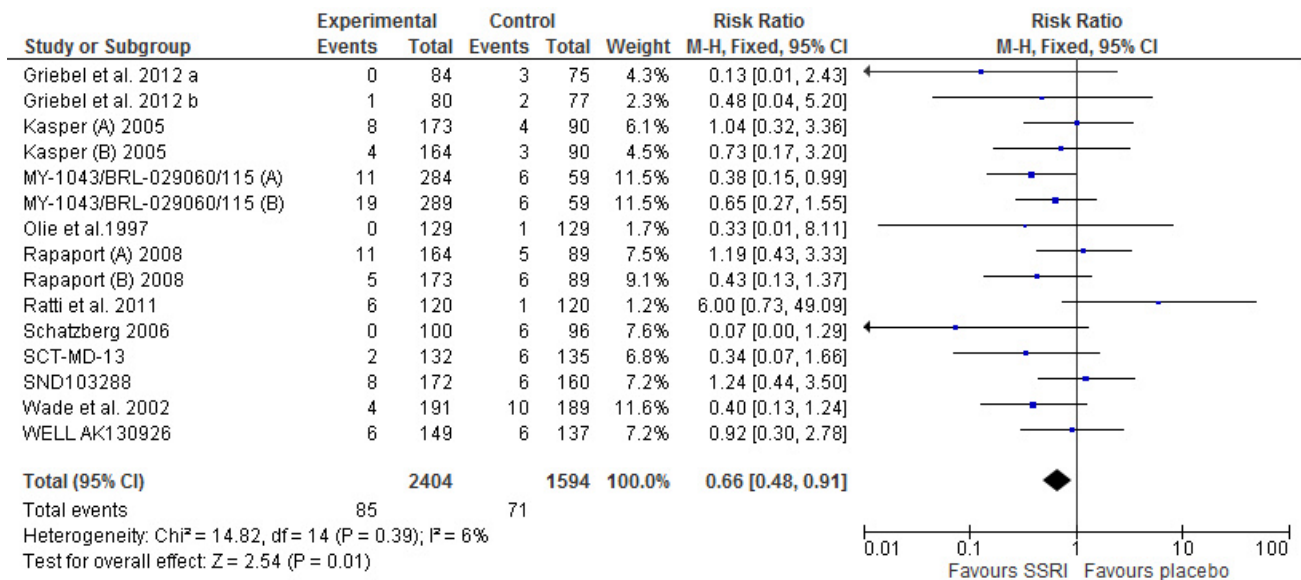

Supplement: Supplementary file 28 — Meta-analysis of back pain. (PDF 153 kb) [file 12888_2016_1173_MOESM28_ESM.pdf]

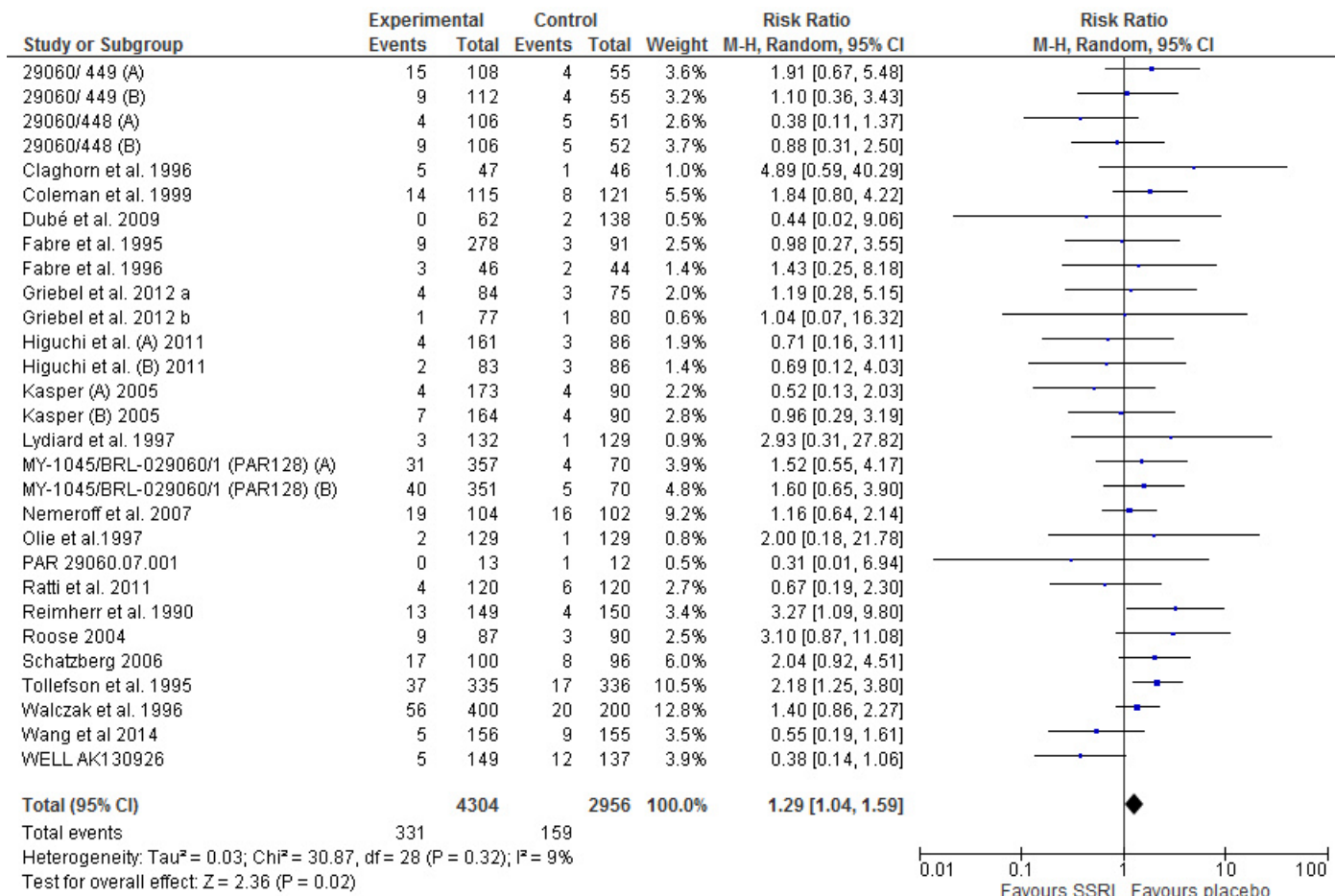

Supplement: Supplementary file 29 — Meta-analysis of headache. [file 12888_2016_1173_MOESM29_ESM.pdf]

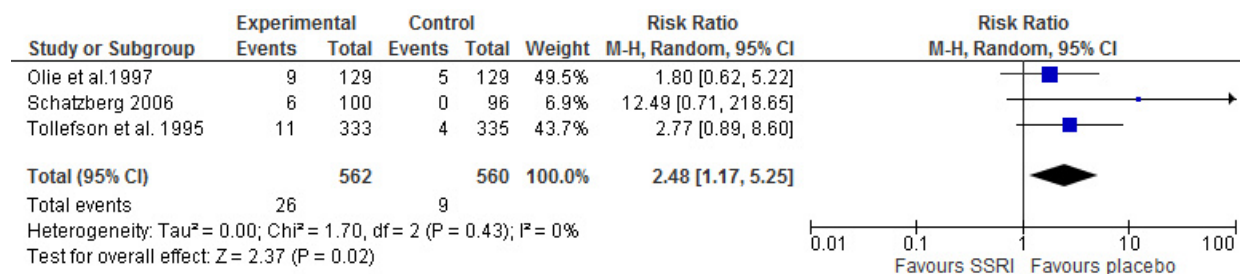

Supplement: Supplementary file 30 — Meta-analysis of dyspepsia. (PDF 226 kb) [file 12888_2016_1173_MOESM30_ESM.pdf]

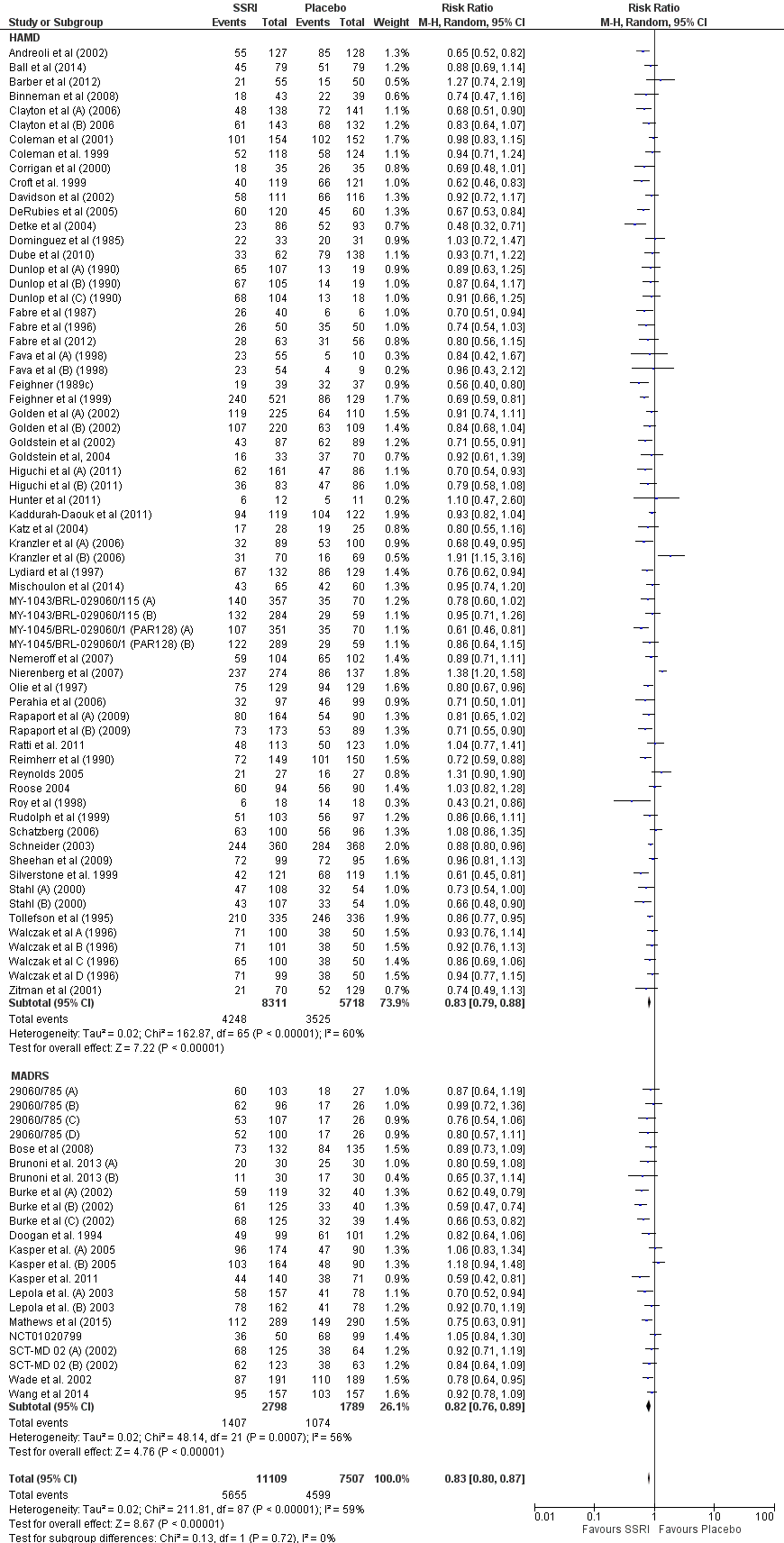

Supplement: Supplementary file 32 — Meta-analysis of no response. (PNG 105 kb) [file 12888_2016_1173_MOESM32_ESM.png]
