# Supplementary material for: Selective serotonin reuptake inhibitors versus placebo in patients with major depressive disorder. A systematic review with meta-analysis and Trial Sequential Analysis
Source: BMC Psychiatry. 2017 Feb 8;17:58. doi: 10.1186/s12888-016-1173-2 (PMC5299662; doi:10.1186/s12888-016-1173-2)
Supplement: Supplementary file 5 — Summary of findings table. (DOCX 72 kb) [file 12888_2016_1173_MOESM5_ESM.docx]

| **Summary of findings table** | | | | | | |
| --- | --- | --- | --- | --- | --- | --- |
| \| **Outcomes** \|  \| \|  \|  \|  \|  \| \| --- \| --- \| --- \| --- \| --- \| --- \| --- \| \|  \|  \| | **Risk with placebo** | **Risk with SSRIs** | **Relative effect (95% CI)** | **№ of participants (trials)** | **Quality of the evidence (GRADE** | **Comments** |
| Hamilton Depression Rating Scale (HDRS) | - | - | The mean HDRS score at end of treatment in the SSRI group was 1.94 (2.50 to -1.3) HDRS points lower than the placebo group | 10,464 (49 trials) | ⊕⊕⊝⊝ Very low | Trial sequential analysis showed that the boundary for harm was crossed |
| Serious adverse events | 22 per 1,000 | 31 (25 to 40) per 1,000 | OR 1.37 (1.08 to 1.75) | 13,299 (44 trials) | ⊕⊕⊝⊝ Very low | Trial sequential analysis showed that the boundary for harm was crossed |
| No remission | 746 per 1,000 | 657 (642 to 679) per 1,000 | RR 0.88 (0.84 to 0.91) | 4214 (34 trials) | ⊕⊕⊝⊝ Very low | Trial sequential analysis showed that the boundary for benefit was crossed |

| ***The risk in the intervention group** (and its 95% confidence interval) is based on the observed risk in the comparison group and the **relative effect** of the intervention (and its 95% CI).  **CI:** Confidence interval; **OR:** Odds ratio; **RR:** Risk ratio; |
| --- |
| **GRADE Working Group grades of evidence** **High quality:** We are very confident that the true effect lies close to that of the estimate of the effect **Moderate quality:** We are moderately confident in the effect estimate: The true effect is likely to be close to the estimate of the effect, but there is a possibility that it is substantially different **Low quality:** Our confidence in the effect estimate is limited: The true effect may be substantially different from the estimate of the effect **Very low quality:** We have very little confidence in the effect estimate: The true effect is likely to be substantially different from the estimate of effect |
