# Supplementary material for: Selective serotonin reuptake inhibitors versus placebo in patients with major depressive disorder. A systematic review with meta-analysis and Trial Sequential Analysis
Source: BMC Psychiatry. 2017 Feb 8;17:58. doi: 10.1186/s12888-016-1173-2 (PMC5299662; doi:10.1186/s12888-016-1173-2)
Supplement: Supplementary file 6 — Summary of the 20 Most Common Adverse Events in the Included Trials. (PDF 37 kb) [file 12888_2016_1173_MOESM6_ESM.pdf]

Event	No. of trials reporting the event	SSRI		Placebo		Relative risk (95% CI)	Number needed to harm (NNH)	P value
		Number of participants with the event	Number of participants randomised	Number of participants with the event	Number of participants randomised			
Abnormal ejaculation	15	183	3236	7	1903	5.43 [3.22, 9.14]	19	P < 0.00001
Tremor	28	301	3502	61	2929	3.16 [2.37, 4.21]	16	P < 0.00001
Anorexia	19	220	2350	42	1680	2.78 [2.03, 3.79]	15	P < 0.00001
Nausea	78	2524	12257	779	8491	2.48 [2.22, 2.77]	9	P < 0.00001
Somnolence	59	1336	10351	345	6674	2.25 [2.00, 2.53]	13	P < 0.00001
Sweating	34	440	5274	124	3478	2.20 [1.80, 2.70]	21	P < 0.00001
Asthenia	23	497	3968	155	2265	1.71 [1.43, 2.04]	18	P < 0.00001
Diarrhoea	58	1458	11056	561	7099	1.66 [1.51, 1.83]	19	P < 0.00001
Constipation	50	606	6698	273	4892	1.60 [1.35, 1.89]	29	P < 0.00001
Insomnia	69	1500	11934	582	7956	1.49 [1.35, 1.64]	19	P < 0.00001
Dizziness	55	849	8900	398	6161	1.39 [1.24, 1.57]	33	P < 0.00001
Dry mouth	73	1376	11303	693	7904	1.37 [1.25, 1.49]	30	P < 0.00001
Libido decreased	8	78	1481	11	1083	3.48 [1.92, 6.32]	24	P < 0.0001
Sexual dysfunction	6	96	719	16	389	2.85 [1.77, 4.59]	11	P = 0.0001
Appetite decreased	8	68	932	24	885	2.63 [1.66, 4.17]	22	P < 0.0001
Fatigue	26	409	5098	153	3545	1.69 [1.32, 2.17]	27	P < 0.0001
Vomiting or upset stomach	20	189	2376	101	2037	1.55 [1.16, 2.08]	34	P = 0.003
Flu syndrome	7	57	1069	19	822	2.13 [1.28, 3.54]	34	P = 0.004
Drowsiness	5	38	253	19	256	1.90 [1.18, 3.04]	14	P = 0.004
Blurred/abnormal vision or dry eyes	17	116	1862	55	1566	1.55 [1.15, 2.10]	37	P = 0.004
Nervousness	22	484	3863	147	2043	1.35 [1.10, 1.66]	19	P = 0.004
Back pain	11	85	2404	71	1594	0.66 [0.48, 0.91]	109	P = 0.01
Headache	72	2386	11085	1427	7805	1.08 [1.01, 1.14]	31	P = 0.02
Dyspepsia	23	331	4304	159	2956	1.29 [1.04, 1.59]	44	P = 0.02
Weight loss	3	26	562	9	560	2.48 [1.17, 5.25]	34	P = 0.02
