# Supplementary material for: Selective serotonin reuptake inhibitors versus placebo in patients with major depressive disorder. A systematic review with meta-analysis and Trial Sequential Analysis
Source: BMC Psychiatry. 2017 Feb 8;17:58. doi: 10.1186/s12888-016-1173-2 (PMC5299662; doi:10.1186/s12888-016-1173-2)

	SSRI		Placebo			Risk Ratio
Study or Subgroup	Events	Total	Events	Total	Weight	M-H, Random, 95% CI
29060/ 449 (A)	24	108	5	55	1.7%	2.44 [0.99, 6.06]
29060/ 449 (B)	25	112	5	55	1.7%	2.46 [0.99, 6.07]
29060/448 (A)	23	106	3	51	1.0%	3.69 [1.16, 11.72]
29060/448 (B)	21	106	3	52	1.0%	3.43 [1.07, 10.99]
29060/785 (A)	14	103	3	27	1.0%	1.22 [0.38, 3.95]
29060/785 (B)	8	96	2	26	0.6%	1.08 [0.24, 4.80]
29060/785 (C)	15	107	2	26	0.7%	1.82 [0.44, 7.48]
29060/785 (D)	20	100	2	26	0.7%	2.60 [0.65, 10.42]
Ball et al (2014)	19	79	16	79	4.0%	1.19 [0.66, 2.14]
Cassano et al. 1986	17	161	2	149	0.7%	7.87 [1.85, 33.47]
Claghorn 1992a	7	36	1	35	0.3%	6.81 [0.88, 52.50]
Claghorn et al. 1996	19	47	4	46	1.4%	4.65 [1.71, 12.62]
Cohn et al. 1991	9	40	7	40	1.8%	1.29 [0.53, 3.12]
Coleman et al. 2001	17	154	6	152	1.7%	2.80 [1.13, 6.90]
Corrigan et al. 2000	3	33	2	34	0.5%	1.55 [0.28, 8.66]
Croft et al. 1999	20	118	7	119	2.1%	2.88 [1.27, 6.56]
Dominguez et al. 1985	8	33	2	31	0.6%	3.76 [0.86, 16.34]
Doogan et al. 1994	2	99	0	101	0.2%	5.10 [0.25, 104.90]
Dunbar et al. 1991	57	240	26	240	7.6%	2.19 [1.43, 3.36]
Fabre 1992	11	39	6	38	1.8%	1.79 [0.73, 4.34]
Fabre et al. 1995	38	278	7	91	2.3%	1.78 [0.82, 3.84]
Fabre et al. 1996	19	46	2	44	0.7%	9.09 [2.25, 36.75]
Feighner et al. 1989b	9	31	1	19	0.4%	5.52 [0.76, 40.17]
Feighner et al. 1999	83	521	5	129	1.8%	4.11 [1.70, 9.93]
Goldstein et al (2002)	7	33	7	70	1.5%	2.12 [0.81, 5.55]
Goldstein et al, 2004	7	87	2	89	0.6%	3.58 [0.76, 16.76]
Griebel et al. 2012 a	5	84	1	75	0.3%	4.46 [0.53, 37.35]
Griebel et al. 2012 b	6	80	2	77	0.6%	2.89 [0.60, 13.87]
Higuchi et al. (A) 2011	16	161	2	86	0.7%	4.27 [1.01, 18.15]
Higuchi et al. (B) 2011	7	83	1	86	0.3%	7.25 [0.91, 57.68]
Itil et al. 1983	11	22	5	22	1.8%	2.20 [0.92, 5.29]
Kasper (A) 2005	4	173	1	90	0.3%	2.08 [0.24, 18.34]
Kasper (B) 2005	0	164	0	90		Not estimable
Kasper et al. 1995	21	110	4	108	1.3%	5.15 [1.83, 14.52]
Lepola et al. (A) 2003	8	155	1	77	0.3%	3.97 [0.51, 31.21]
Lepola et al. (B) 2003	5	160	1	77	0.3%	2.41 [0.29, 20.24]
LVM-MD-06	6	79	4	93	0.9%	1.77 [0.52, 6.04]
Lydiard et al. 1997	14	132	7	129	1.8%	1.95 [0.82, 4.69]
MY-1043/BRL-029060/115 (A)	69	284	4	59	1.5%	3.58 [1.36, 9.44]
MY-1043/BRL-029060/115 (B)	51	289	5	59	1.8%	2.08 [0.87, 4.99]
MY-1045/BRL-029060/1 (PAR128) (A)	84	357	5	70	1.9%	3.29 [1.39, 7.82]
MY-1045/BRL-029060/1 (PAR128) (B)	58	351	5	70	1.8%	2.31 [0.96, 5.56]
NCT00668525 (A)	17	322	2	109	0.7%	2.88 [0.68, 12.25]
NCT00668525 (B)	26	324	2	109	0.7%	4.37 [1.06, 18.13]
NCT01020799	3	50	5	99	0.7%	1.19 [0.30, 4.77]
NCT01473381	22	282	10	281	2.6%	2.19 [1.06, 4.54]
Nierenberg et al (2007)	18	274	5	137	1.5%	1.80 [0.68, 4.75]
Olie et al.1997	5	129	0	129	0.2%	11.00 [0.61, 196.91]
PAR 29060.07.001	5	13	2	12	0.7%	2.31 [0.55, 9.74]
Perahia et al (2006)	5	97	0	99	0.2%	11.22 [0.63, 200.28]
Peselow et al. 1989a	3	40	2	42	0.5%	1.57 [0.28, 8.94]
Rapaport (A) 2008	8	164	3	89	0.8%	1.45 [0.39, 5.32]
Rapaport (B) 2008	15	173	2	89	0.7%	3.86 [0.90, 16.50]
Ratti et al. 2011	7	120	7	120	1.3%	1.00 [0.36, 2.76]
Reimherr et al. 1990	29	149	18	150	4.7%	1.62 [0.94, 2.79]
Rickels et al. 1992	13	55	4	56	1.2%	3.31 [1.15, 9.52]
Roose 2004	5	87	4	90	0.8%	1.29 [0.36, 4.66]
Rudolph et al. 1999	12	103	6	98	1.6%	1.90 [0.74, 4.87]
Schatzberg 2006	6	100	1	96	0.3%	5.76 [0.71, 46.96]
Schneider 2003	38	371	16	376	4.3%	2.41 [1.37, 4.24]
SCT-MD 02 (A) 2002	13	125	3	64	0.9%	2.22 [0.66, 7.51]
SCT-MD 02 (B) 2002	9	123	3	63	0.9%	1.54 [0.43, 5.48]
SCT-MD-13	12	132	5	135	1.4%	2.45 [0.89, 6.78]
SCT-MD-27 (A)	14	136	2	67	0.7%	3.45 [0.81, 14.74]
SCT-MD-27 (B)	16	138	2	68	0.7%	3.94 [0.93, 16.65]
Shrivastava et al. 1992	10	40	6	40	1.7%	1.67 [0.67, 4.15]
Silverstone et al. 1999	17	121	7	119	2.0%	2.39 [1.03, 5.55]
Smith et al.1992	7	39	3	38	0.9%	2.27 [0.63, 8.15]
SND103288	17	172	11	160	2.6%	1.44 [0.69, 2.97]
Wakelin 1986	5	32	2	14	0.6%	1.09 [0.24, 4.97]
Walczak et al. 1996	77	400	16	200	5.3%	2.41 [1.44, 4.01]
Wang et al 2014	13	156	6	155	1.6%	2.15 [0.84, 5.52]
WELL AK130926	11	149	8	137	1.8%	1.26 [0.52, 3.05]
WELL AK130927	11	138	6	141	1.5%	1.87 [0.71, 4.92]
**Total (95% CI)**		**10351**		**6674**	**100.0%**	**2.25 [2.00, 2.53]**
Total events	1336		345			

Heterogeneity: $Tau^2 = 0.00$; $Chi^2 = 50.26$, df = 72 (P = 0.98); $I^2 = 0\%$

Test for overall effect: Z = 13.43 (P < 0.00001)

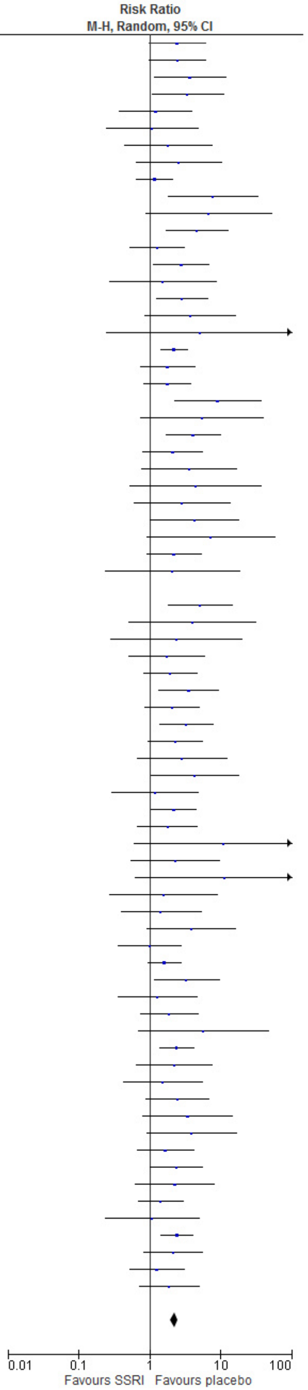

Supplement: Supplementary file 11 — Meta-analysis of somnolence. (PDF 459 kb) [file 12888_2016_1173_MOESM11_ESM.pdf]
