# Supplementary material for: Selective serotonin reuptake inhibitors versus placebo in patients with major depressive disorder. A systematic review with meta-analysis and Trial Sequential Analysis
Source: BMC Psychiatry. 2017 Feb 8;17:58. doi: 10.1186/s12888-016-1173-2 (PMC5299662; doi:10.1186/s12888-016-1173-2)

Study or Subgroup	SSRI Events	SSRI Total	Placebo Events	Placebo Total	Weight	Risk Ratio M-H, Random, 95% CI
29060/ 449 (A)	9	108	3	55	1.5%	1.53 [0.43, 5.42]
29060/ 449 (B)	18	112	3	55	1.7%	2.95 [0.91, 9.58]
29060/448 (A)	13	106	2	51	1.2%	3.13 [0.73, 13.34]
29060/448 (B)	8	106	1	52	0.6%	3.92 [0.50, 30.55]
Byerley et al. 1988	9	32	3	29	1.6%	2.72 [0.81, 9.08]
Cassano et al. 1986	13	161	5	149	2.2%	2.41 [0.88, 6.59]
Claghorn 1992a	8	36	0	35	0.4%	16.54 [0.99, 276.12]
Claghorn et al. 1996	6	47	4	46	1.7%	1.47 [0.44, 4.86]
Cohn et al. 1985	5	54	3	58	1.3%	1.79 [0.45, 7.13]
Cohn et al. 1991	3	40	6	40	1.4%	0.50 [0.13, 1.86]
Dominguez et al. 1985	6	33	2	31	1.1%	2.82 [0.61, 12.93]
Doogan et al. 1994	1	99	1	101	0.4%	1.02 [0.06, 16.09]
Dubé et al. 2009	1	62	7	138	0.6%	0.32 [0.04, 2.53]
Dunbar et al. 1991	38	240	29	240	5.9%	1.31 [0.84, 2.05]
Edwards et al. 1993	1	21	0	20	0.3%	2.86 [0.12, 66.44]
Fabre 1992	8	39	7	38	2.6%	1.11 [0.45, 2.77]
Fabre et al. 1995	12	278	0	91	0.3%	8.24 [0.49, 137.87]
Fabre et al. 1996	5	46	0	44	0.3%	10.53 [0.60, 185.02]
Feighner et al. 1989a	5	51	3	48	1.3%	1.57 [0.40, 6.21]
Feighner et al. 1989b	5	31	2	19	1.1%	1.53 [0.33, 7.13]
Goldstein et al (2002)	5	33	4	70	1.6%	2.65 [0.76, 9.24]
Goldstein et al, 2004	12	87	3	89	1.6%	4.09 [1.20, 14.00]
Griebel et al. 2012 b	5	80	4	77	1.5%	1.20 [0.34, 4.31]
Higuchi et al. (A) 2011	17	161	3	86	1.7%	3.03 [0.91, 10.04]
Higuchi et al. (B) 2011	7	83	2	86	1.1%	3.63 [0.78, 16.95]
Kasper (A) 2005	2	173	4	90	0.9%	0.26 [0.05, 1.39]
Kasper (B) 2005	7	164	4	90	1.7%	0.96 [0.29, 3.19]
Kasper et al. 1995	23	110	8	108	3.3%	2.82 [1.32, 6.03]
Kranzler et al. (A) 2006	16	89	4	100	2.0%	4.49 [1.56, 12.94]
Kranzler et al. (B) 2006	15	70	4	69	2.1%	3.70 [1.29, 10.58]
Learned et al. 2012	14	166	8	156	2.9%	1.64 [0.71, 3.81]
LVM-MD-06	3	79	3	93	1.0%	1.18 [0.24, 5.67]
Lydiard et al. 1997	5	132	2	129	1.0%	2.44 [0.48, 12.37]
McGrath et al. 2000	8	49	2	52	1.1%	4.24 [0.95, 19.02]
MY-1045/BRL-029060/1 (PAR128) (A)	41	357	6	70	3.0%	1.34 [0.59, 3.03]
MY-1045/BRL-029060/1 (PAR128) (B)	21	351	6	70	2.7%	0.70 [0.29, 1.67]
Nemeroff et al. 2007	2	104	5	102	1.0%	0.39 [0.08, 1.98]
Nierenberg et al (2007)	16	274	8	137	3.0%	1.00 [0.44, 2.28]
Norton et al. 1984	9	35	1	25	0.7%	6.43 [0.87, 47.56]
PAR 29060.07.001	2	13	1	12	0.5%	1.85 [0.19, 17.84]
Perahia et al (2006)	2	97	5	99	1.0%	0.41 [0.08, 2.05]
Peselow et al. 1989a	9	40	7	42	2.7%	1.35 [0.56, 3.28]
Rapaport (A) 2008	14	164	7	89	2.7%	1.09 [0.45, 2.59]
Rapaport (B) 2008	20	173	7	89	3.0%	1.47 [0.65, 3.34]
Ratti et al. 2011	9	120	4	120	1.8%	2.25 [0.71, 7.11]
Reimherr et al. 1990	17	149	10	150	3.4%	1.71 [0.81, 3.61]
Roose 2004	10	87	4	90	1.9%	2.59 [0.84, 7.94]
Schatzberg 2006	10	100	4	96	1.8%	2.40 [0.78, 7.39]
SCT-MD-13	10	132	5	135	2.1%	2.05 [0.72, 5.82]
Sheehan et al. 2009	8	99	11	95	2.8%	0.70 [0.29, 1.66]
Shrivastava et al. 1992	7	40	1	40	0.6%	7.00 [0.90, 54.32]
Trivedi et al. (A) 2004	7	156	3	75	1.4%	1.12 [0.30, 4.22]
Trivedi et al. (B) 2004	12	154	3	74	1.6%	1.92 [0.56, 6.60]
Wakelin 1986	8	32	1	14	0.7%	3.50 [0.48, 25.39]
Walczak et al. 1996	37	400	12	200	4.2%	1.54 [0.82, 2.89]
Wang et al 2014	13	156	7	155	2.7%	1.85 [0.76, 4.50]
WELL AK130926	5	149	12	137	2.2%	0.38 [0.14, 1.06]
WELL AK130927	4	138	7	141	1.6%	0.58 [0.17, 1.95]
**Total (95% CI)**		**6698**		**4892**	**100.0%**	**1.60 [1.35, 1.89]**
Total events	606		273			

Heterogeneity: $Tau^2 = 0.08$; $Chi^2 = 70.26$, df = 57 (P = 0.11); $I^2 = 19\%$

Test for overall effect: Z = 5.42 (P < 0.00001)

Risk Ratio
M-H, Random, 95% CI

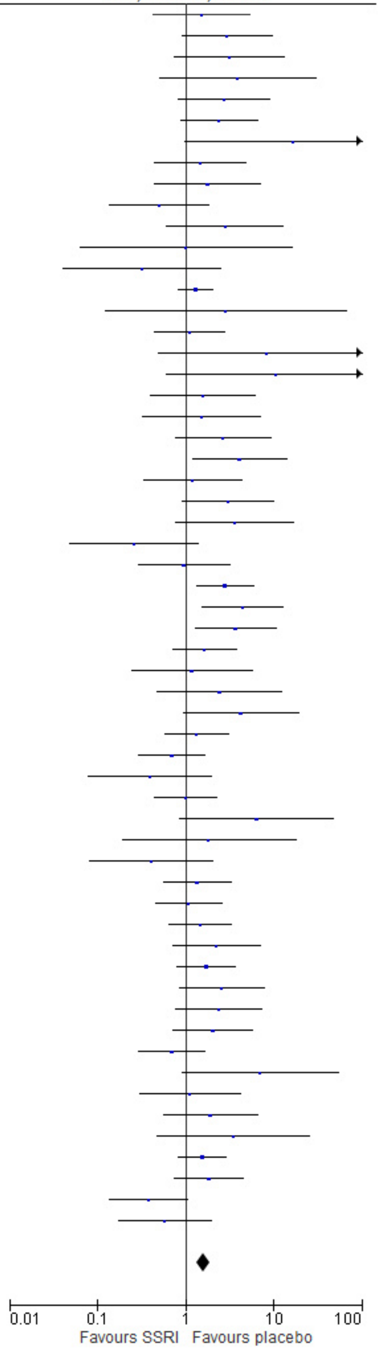

Supplement: Supplementary file 15 — Meta-analysis of constipation. (PDF 369 kb) [file 12888_2016_1173_MOESM15_ESM.pdf]
